# Insights into Lignan Composition and Biosynthesis in Stinging Nettle (*Urtica dioica* L.)

**DOI:** 10.3390/molecules24213863

**Published:** 2019-10-26

**Authors:** Xuan Xu, Cédric Guignard, Jenny Renaut, Jean-Francois Hausman, Edoardo Gatti, Stefano Predieri, Gea Guerriero

**Affiliations:** 1 Environmental Research and Innovation (ERIN) Department, Luxembourg Institute of Science and Technology (LIST), L-4362 Esch/Alzette, Luxembourg; xuan.xu@list.lu (X.X.); cedric.guignard@list.lu (C.G.); jenny.renaut@list.lu (J.R.); jean-francois.hausman@list.lu (J.-F.H.); 2 Institute of Bioeconomy (IBE), National Research Council, I-40129 Bologna, Italy; e.gatti@ibimet.cnr.it (E.G.); s.predieri@ibimet.cnr.it (S.P.)

**Keywords:** lignan, dirigent protein, pinoresinol-lariciresinol reductase, gene expression, bioinformatics, *Urtica dioica* L.

## Abstract

Stinging nettle (*Urtica dioica* L.) has been used as herbal medicine to treat various ailments since ancient times. The biological activity of nettle is chiefly attributed to a large group of phenylpropanoid dimers, namely lignans. Despite the pharmacological importance of nettle lignans, there are no studies addressing lignan biosynthesis in this plant. We herein identified 14 genes encoding dirigent proteins (*UdDIR*s) and 3 pinoresinol-lariciresinol reductase genes (*UdPLR*s) in nettle, which are two gene families known to be associated with lignan biosynthesis. Expression profiling of these genes on different organs/tissues revealed a specific expression pattern. Particularly, *UdDIR7*, *12* and *13* displayed a remarkable high expression in the top internode, fibre tissues of bottom internodes and roots, respectively. The relatively high expression of *UdPLR1* and *UdPLR2* in the young internodes, core tissue of bottom internode and roots is consistent with the high accumulation of lariciresinol and secoisolariciresinol in these tissues. Lignan quantification showed a high abundance of pinoresinol in roots and pinoresinol diglucosides in young internodes and leaves. This study sheds light on lignan composition and biosynthesis in nettle, providing a good basis for further functional analysis of *DIR*s and *PLR*s and, ultimately, engineering lignan metabolism *in planta* and in cell cultures.

## 1. Introduction

Lignans, a large group of phenylpropanoid dimers, are widely distributed across the plant kingdom. Their primary biological function *in planta* is supposed to be associated with plant defence [1,2], particularly in response to pathogen attack [3]. In addition, lignans have received great interest due to their numerous beneficial effects in mammals, such as antihypertensive, antitumor, hepatoprotective, insecticidal, estrogenic, sedative and antioxidant activities [4]. For centuries, plants with a high lignan content have been used as an important and popular herbal medicine in the Eastern World [5]; one of these plants is stinging nettle (*Urtica dioica* L.), a perennial dioecious plant spread throughout the temperate zones of the world [6,7,8].

Stinging nettle is commonly considered an invasive weed; nevertheless, the leaf and root of this herbaceous plant have been widely used to treat many ailments including arthritis, rheumatism, hypertension, eczema, allergic rhinitis and muscular paralysis [9,10,11,12,13]. The extracts of nettle roots have been used in the treatment of benign prostatic hyperplasia and prostatic disease [8]. It was reported that the beneficial effect of extracts was partially attributed to lignans, which can bind to sex hormone-binding globulin, thus inhibiting the interaction with the receptor [7].

Despite the beneficial effects of nettle lignans, only a few studies reported the lignan composition and content in the roots of nettle [14,15]. Studies on lignan composition in different tissues and organs of nettle would provide additional knowledge that can be exploited to devise biotechnological strategies aimed at increasing lignan production in *U. dioica*.

Lignans display considerable diversity in their basic chemical structure due to the varying degree of oxidation and substitution of their aromatic moieties [16,17]. In addition, the enantiomeric composition of lignans differs substantially among plant species, as well as developmental stages and different organs within the same plant [18,19]. This heterogeneity is mainly determined through reactions mediated by two key players, namely dirigent proteins (DIRs) and pinoresinol-lariciresinol reductase (PLR) [19].

More specifically, DIRs partake in the initial step of lignan biosynthesis, where pinoresinol (PINO) is formed via stereospecific coupling of two coniferyl alcohols [20,21]. A large number of DIRs were identified in different plant species and further clustered into six distinct subfamilies (i.e., a, b/d, c, e, f and g) using phylogenetic analyses [22]. The physiological role of DIRs is versatile, as they are associated with a wide range of physiological processes besides lignan production [23], such as lignification [24,25] and (a)biotic stress response [22,26,27]. Their biochemical function can be inferred, to some extent, based on the phylogenetic clustering, especially for those DIRs involved in lignan production, which are clustered together in subfamily-a [22].

PLRs, that are NADPH-dependent bifunctional proteins, catalyze sequential reduction of PINO to lariciresinol (LARI) and then secoisolariciresinol (SECO). The substrate-selective and enantiospecific features of PLRs result in the stereochemical diversity of lignans in different plant species and even different organs of the same plant. For example, LuPLR1 (*Linum usitatissimum*) and TpPLR1 (*Thuja plicata*) reduce (−)-PINO into (+)-SECO via (–)-LARI, while LuPLR2 and TpPLR2 convert (+)-PINO into (−)-SECO via (+)-LARI [28,29]. Moreover, *LuPLR2* was transcriptionally active only in leaves and stems, whereas both *LuPLR1* and *LuPLR2* were expressed in seeds, a finding explaining the distinct enantiomeric composition of lignans in different organs [29].

So far, to the best of our knowledge, there is no study on the identification of lignan biosynthetic genes in stinging nettle, nor on their gene expression profiling in different tissues. Yet, our recently published data on the transcriptome of nettle “clone 13” provides us with a good starting point [30]. In the work presented herein, we identified nettle members of *DIR*s and *PLR*s and conducted phylogenetic analyses, with the goal of enriching the knowledge on lignan biosynthesis in nettle. Gene expression profiling was coupled to lignan identification via a targeted metabolite approach. This work, for the first time, provides insights into lignan biosynthesis in this multi-purpose, yet neglected plant and paves the way to follow-up studies aiming at modulating lignan metabolism and ultimately improving lignan production *in planta*, as well as in nettle cell cultures.

## 2. Results

### 2.1. Identification of UdDIRs

**Figure 1:**
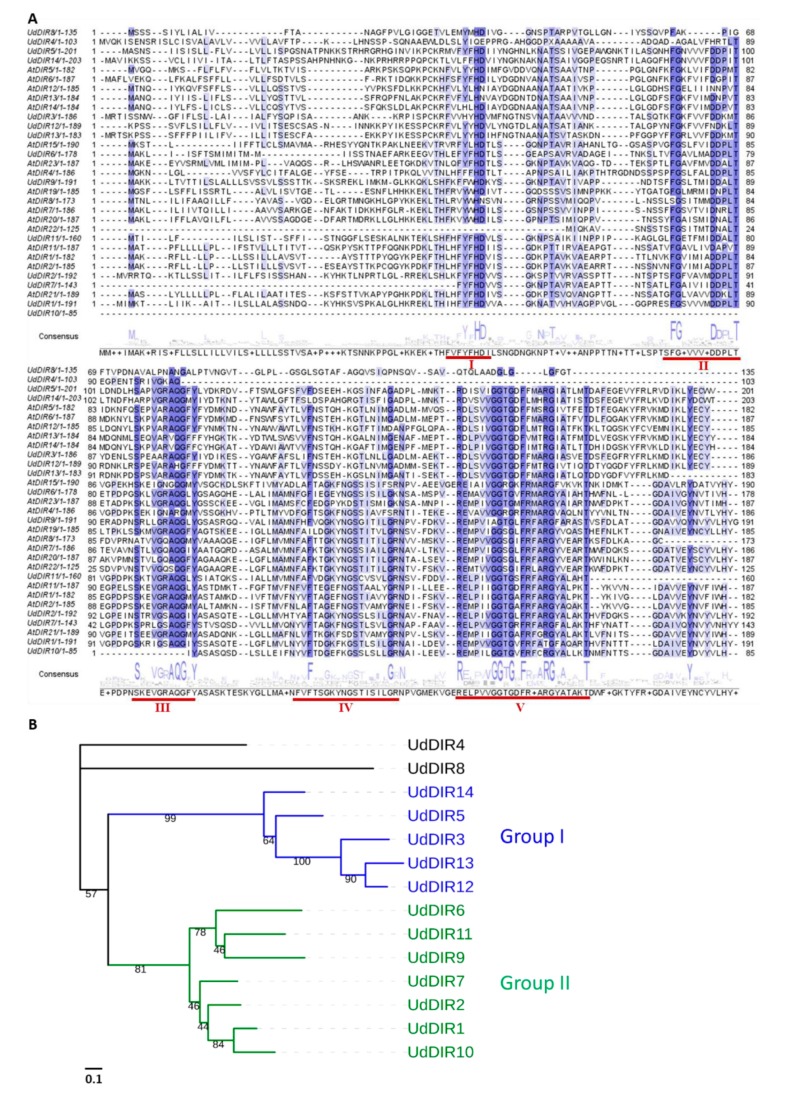
(**A**) Amino acid sequence alignment of DIRs from *U. dioica* (Ud) and selected *A. thaliana* (At) sequences. The alignment was generated with CLUSTAL-Ω and the conserved residues were highlighted using Jalview. Five conserved motifs (I–V) reported previously in [22] were identified in the amino acid sequences of UdDIRs and are underlined in red. (**B**) Phylogenetic analysis of UdDIRs. The tree was built by the maximum likelihood method with 1000 bootstraps. The scale bar indicates 0.1 amino acid substitutions per site.

The previously established *U. dioica de novo* transcriptome was used to identify *UdDIR* genes [30]. Sixteen contigs were annotated as *DIR*s using Blast2GO against the *A. thaliana* and Viridiplantae database. BLASTN and BLASTX analyses against nettle leaf transcriptome at oneKP database were further carried out to examine and verify the obtained contigs. The sequence of some contigs was reconstructed to obtain the full length. A total of 14 *DIR*s were ultimately identified in *U. dioica* and 8 contain a full-length predicted open reading frame (ORF), ranging from 178 (UdDIR6) to 203 (UdDIR14) amino acids (Table 1). *UdDIR* nucleotide and protein sequences are listed in Text S1. We performed the alignment on the protein sequences of UdDIRs and selected AtDIRs. The five conserved motifs described previously [22] were identified in the sequences of UdDIRs (Figure 1A).

To investigate the similarities and divergences of the 14 UdDIRs, a multiple alignment of amino acid sequences was used to build a maximum likelihood tree (Figure 1B). UdDIRs were mainly separated into two main groups. UdDIR14/5/3/13/12 clustered into Group I, while UdDIR1/2/6/7/9/10/11 into Group II. UdDIR4 and UdDIR8 did not cluster into any group, indicative of the high sequence divergence of these two DIRs as compared to the others.

**Figure 2:**
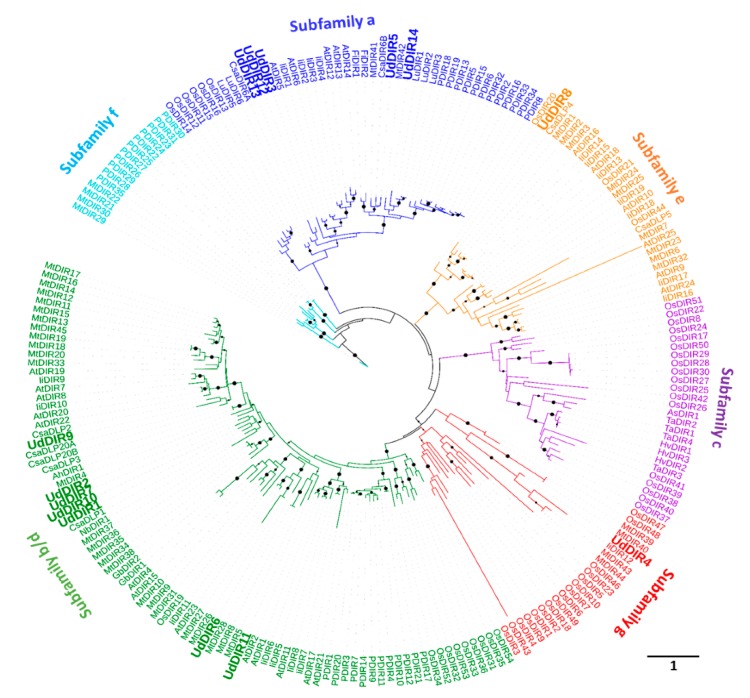
Phylogenetic analysis of DIRs from various plant species. *M. truncatula* (Mt), *A. thaliana* (At), *C. sativa* (Csa), *L. usitatissimum* (Lu), *P. sitchensis* (P), *O. sativa* (Os), *A. hypogaea* (Ah), *A. stolonifera* (As), *F. x intermedia* (Fi), *G. barbadense* (Gb), *N. benthamiana* (Nb), *T. aestivum* (Ta), *H. vulgare* (Hv) and *I. indigotica* (Li). The tree was constructed by the maximum likelihood method with 1000 bootstraps. Bootstrap values are indicated for nodes with support higher than 90% (black circles; the bigger the circle, the higher the value). The scale bar indicates 1 amino acid substitutions per site.

To further understand the evolutionary relationships of DIRs among *U. dioica* and other plant species, an unrooted phylogenetic tree was constructed using 218 DIRs protein sequences from different plant species. As shown in Figure 2, all DIRs were classified into six subfamilies based on the classification of [22], with subfamily-c consisting of only angiosperm monocot DIRs, as previously reported [22]. UdDIRs from Group I (5 DIRs) and Group II (7 DIRs) were assigned to subfamily-a and b/d, respectively. UdDIR8 and UdDIR4 clustered into subfamily-e and g, respectively. Interestingly, the majority of UdDIRs clustered closely with DIRs derived from *C. sativa* and *L. usitatissimum*, suggesting a phylogenetic relatedness of DIRs among fibre crops. For example, in the subfamily-a, a grouping was observed for UdDIR12, UdDIR13 and CsaDIR6A, as well as for UdDIR14 and LuDIR1/2/3.

### 2.2. Identification of UdPLRs

The same approach described above was used to obtain *PLR* genes in *U. dioica.* Three genes encoding *PLR* were identified (nucleotide and protein sequences are listed in Text S1). Among them, UdPLR2/3 contain a complete ORF sequence with 312 and 309 amino acids, respectively (Table 1).

**Figure 3:**
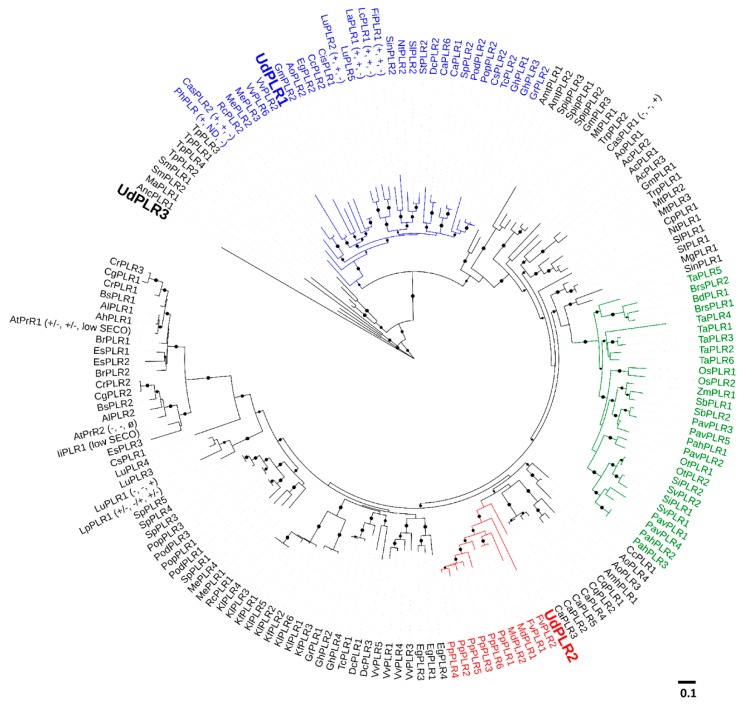
Phylogenetic tree of PLRs from different plant species. The tree was built by the maximum likelihood method with 1000 bootstraps replicates. Bootstrap values > 80% are displayed with black circles (the bigger the circle, the higher the value). Enantiospecificity of characterized PLRs are shown in brackets in the order of (PINO, LARI, SECO), + and − represent two different enantiomeric configurations. Ø refers to “not detected”. ND refers to “not determined”. The cluster of monocots are in green. The additional details of each protein see Table S2. The scale bar indicates 0.1 amino acid substitutions per site.

It has been reported that PLRs display different affinity and enantiospecificity for the substrates (i.e., PINO and LARI enantiomers), which results in the complexity of the action of PLRs and consequently the difficulties in understanding their catalytic function. The foregoing phylogenetic analyses demonstrated that PLRs with similar catalytic activity clustered together [18,31]. In order to shed some light on the function of UdPLRs, a phylogenetic tree was constructed using 170 PLRs full-length protein sequences from 73 plant species, including 12 PLRs that were characterized for their enantiomeric selectivity (Figure 3). The generated tree illustrates that UdPLR1 clusters together with PLRs preferring (+)-PINO and (+)-LARI to form (–)-SECO, namely LuPLR2 [32], LaPLR1 [32], LcPLR1 [33], FiPLR1 [34] and PhPLR [35,36]. Moreover, UdPLR2 is distributed in the same cluster with PLRs from *Prunus persica* (Pp), *Malus domestica* (Md) and *Fragaria vesca* (Fv), which all belong to the order Rosales. However, no PLR in this cluster has been functionally characterized so far. UdPLR3 does not branch together with other PLRs, which is indicative of low similarities in the protein sequence when comparing this gene with other *PLR*s. An analogous result was obtained when the phylogenetic tree was built using UdDIRs and PLRs that were characterized by their enantiospecificity (Figure S1).

**Figure 4:**
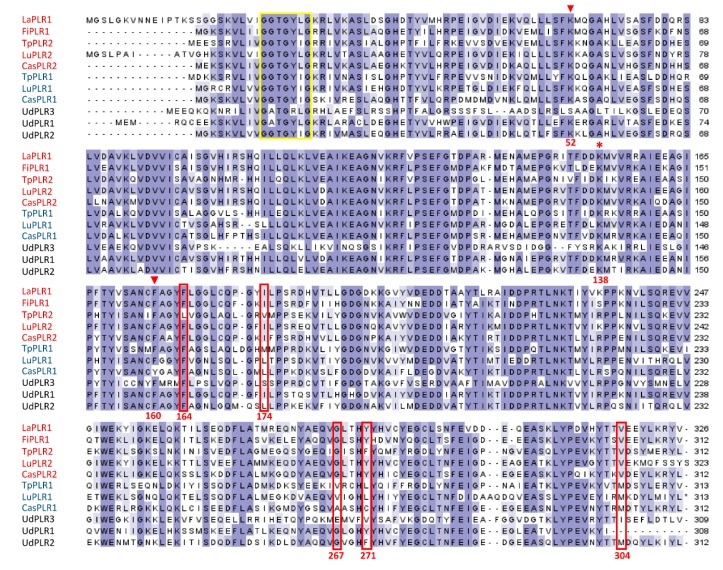
Multiple sequence alignment of UdPLRs and functionally characterised plant PLR proteins. *U. dioica* (Ud), *L. usitatissimum* (Lu), *F. x intermedia* (Fi), *L. album* (La), *T. plicata* (Tp) and *Camellia sinensis* (Cas). PLRs with specificity to form (–)-SECO and (+)-SECO are marked in red and blue, respectively. The conserved motif ‘‘GxxGxxG’’ of the NADPH binding domain is enclosed in the yellow frame. The asterisk indicates amino acid K138 that is involved in general base catalysis [37]. Amino acids that are involved in the enantiospecificity are enclosed with red boxes [31,32,37]. K52 and F160 are associated with the stabilisation of 2’-phosphate group of NADPH and the nicotine amide ring [31,37] and are indicated with triangles. The numbering of amino acids is based on the sequence of UdPLR2.

It was shown that the enantiospecificity of PLRs could be determined by certain amino acids [31,37]. In light of this observation, we conducted a multiple sequence alignment using amino acid sequences of UdPLRs and others catalysing opposite enantiospecific conversions. As shown in Figure 4, similarly to other PLRs, all UdPLRs contained K138, which is associated with the general base catalysis and the NAD(P)H-binding motif “GxxGxxG”. The stabilization of 2’-phosphate group of NADPH and nicotine amide ring was attributed to two sites, namely K52 and F160. The latter was observed in all UdPLR sequences, while the former residue was absent in UdPLR3. Previous studies revealed that some amino acids in PLRs are conservative and discriminative with respect to their enantiospecificity, such as residue 164, 174, 267 and 271. Interestingly, these residues in UdPLR1 were consistent with the ones of PLRs that convert (+)-PINO to (–)-SECO via (+)-LARI, namely LaPLR1, FiPLR1, TpPLR2, LuPLR2 and CasPLR2.

### 2.3. Targeted Quantification of Lignans in Different Tissues of Stinging Nettle

**Figure 5:**
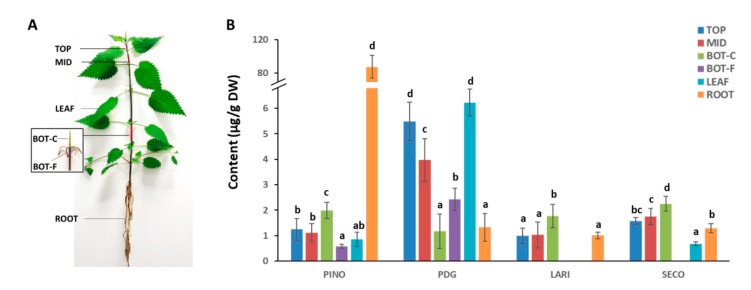
(**A**) Tissues and organs collected for analyses. TOP, MID, BOT-C, BOT-F, LEAF and ROOT represent the top and middle internode, core and cortical tissue of bottom internode, leaves and roots, respectively. (**B**) Content (in µg/g DW) of each lignan in different tissues. The targeted quantification of pinoresinol (PINO), pinoresinol diglucoside (PDG), lariciresinol (LARI), secoisolariciresinol (SECO) was performed using LC-HRMS. Error bars represent the standard deviation calculated from four independent biological replicates and two technical replicates. Significant differences among groups were analysed using one-way ANOVA followed by Tukey’s post-hoc test and are indicated with different letters.

To understand lignan composition in different tissues, we quantified the six most common lignans, namely pinoresinol (PINO), lariciresinol (LARI), secoisolariciresinol (SECO), matairesinol (MATA), pinoresinol diglucoside (PDG) and secoisolariciresinol diglucoside (SDG) in the young internodes (TOP and MID), core and cortical tissues of old internodes (BOT-C and BOT-F), leaves and roots (Figure 5A). SDG and MATA were not detected in any of the studied tissue, possibly due to their very low abundance.

As can be seen in Figure 5B, the accumulation of four lignans varied substantially among different tissues/organs. More specifically, TOP and MID internodes displayed a similar composition, in which the predominant lignan was PDG (5.49 ± 0.76 and 3.97 ± 0.85 µg/g DW in TOP and MID, respectively). In the BOT-C, four lignans were present without a significant difference in the amount. Neither LARI nor SECO were detected in BOT-F, while PDG showed a 4-fold higher amount (2.43 ± 0.44 µg/g DW) with respect to PINO (0.58 ± 0.08 µg/g DW). The predominant lignan in LEAF was PDG (6.23 ± 0.52 µg/g DW), showing a comparable amount as compared to TOP, while no LARI was detected in LEAF. Strikingly, ROOT displayed a remarkably high amount of PINO (87.19 ± 13.73 µg/g DW), which was > 30-fold higher with respect to BOT-F and > 50-fold higher as compared to all other tissues (i.e., TOP, MID, BOT-C and ROOT). It is interesting to note that PINO and PDG accumulated in different tissues and organs in an opposite fashion; tissues/organs with high PINO content have low PDG content and vice versa.

### 2.4. Gene Expression Analysis of DIRs and PLRs in Different Tissues

**Figure 6:**
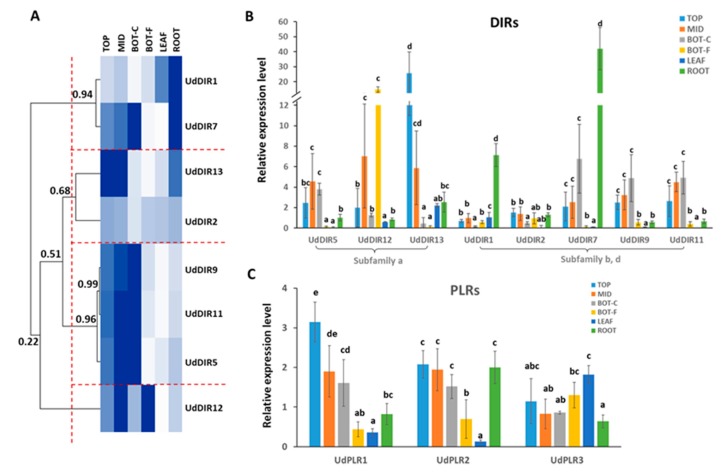
(**A**) Hierarchical clustering of *UdDIR*s expression profiles in different organs and tissues. The correlation coefficient of each cluster is indicated on the branch. Four expression patterns are obtained using Pearson coefficient 0.68 as the threshold and are indicated with red dashed lines. Relative expression of *DIR*s (**B**) and *PLR*s (**C**) in different tissues. TOP, top internode; MID, middle internode; BOT-C, core tissue of bottom internode; BOT-F, cortical tissue of bottom internode. Standard deviation was calculated from the values of four biological replicates. A one-way ANOVA with Tukey’s post-hoc test was used to determine statistically significant differences among the groups, which are indicated with different letters.

To provide further insight into the possible roles of *U. dioica* DIRs and PLRs, we investigated their gene expression in different tissues. The RT-qPCR analysis was carried out on eight *UdDIR*s that were differentially expressed in different internodes and tissues of nettle stem based on previously published RNA-Seq data. The RPKM value of *UdDIR*s and *UdPLR*s in different tissues are given in Table S3 [30]. We reasoned that these *UdDIR*s, rather than those showing a constant expression level, would be the best targets to understand, at the gene level, the differences in the abundance of lignans in different tissues and organs. Of these *UdDIR*s, three genes (i.e., *UdDIR5/12/13*) clustered in subfamily-a and five (i.e., *UdDIR1/2/7/9/11*) were assigned to subfamily-b/d (Figure 1). All genes showed distinct expression patterns over different tissues/organs and interestingly, some genes showed an exceedingly high expression in certain tissues (Figure 6A,B). As shown in the heat map hierarchical clustering of *UdDIR*s expression profiles, four unique expression patterns can be identified by setting 0.68 as the threshold value for the correlation coefficient. These patterns were characterised by those genes that were highly expressed in (1) ROOT (*UdDIR1/7*), (2) young internodes at the TOP and MID (*UdDIR13* and *UdDIR2*), (3) both young internodes and BOT-C (*UdDIR5/9/11*) and (4) BOT-F (*UdDIR12*), respectively (Figure 6A).

More specifically, for *UdDIR1*, while an extremely low expression was observed in BOT-F and LEAF, its expression level showed over 17-fold higher value in ROOT with respect to young internodes (TOP and MID) and 6-fold higher abundance as compared to the BOT-C (Figure 6B). *UdDIR13* was predominantly expressed in the TOP (FC TOP vs. MID and ROOT > 4 and 10, respectively), with low expression in BOT tissue and LEAF. The expression level of *UdDIR12* displayed a sharp peak in the BOT-F. All other *UdDIR*s were nevertheless expressed at low levels in BOT-F with respect to other tissues.

Concerning *PLR*s, while *UdPLR3* showed a comparable expression level in different tissues, *UdPLR1* and *UdPLR2* both displayed a significantly low expression in BOT-F and LEAF, as compared to the other tissues (Figure 6C).

## 3. Discussion

**Figure 7:**
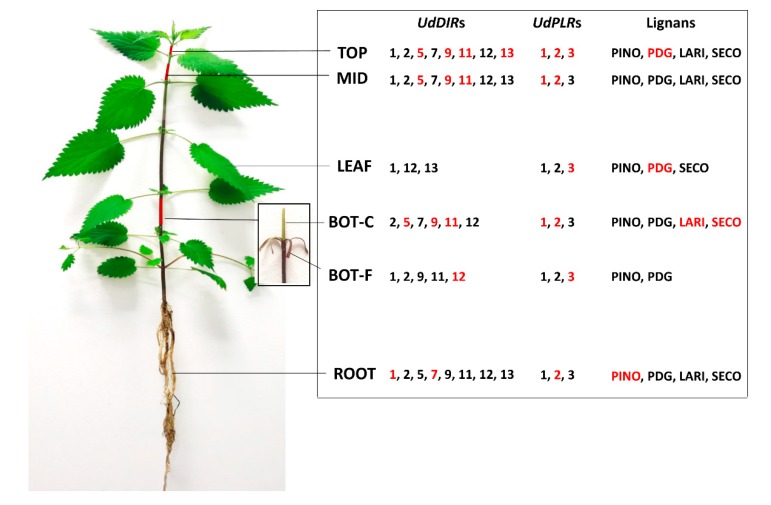
Drawing summarizing tissue/organ-specific gene expression and the lignan classes detected. Genes that are highly expressed in the specific tissue are indicated in red, as well as the highly abundant lignans. PINO, pinoresinol; PDG, pinoresinol diglucoside; LARI, lariciresinol; SECO, secoisolariciresinol.

In this study, with the aim of improving the knowledge on lignan biosynthesis in *U. dioica*, nettle members of *DIR*s and *PLR*s were identified and analysed. Interestingly, the expression of these genes, as well as the lignan profile, showed organ/tissue-specific patterns, which is summarized in Figure 7.

Mining of the *U. dioica* transcriptome revealed a family of (at least) 14 DIRs, showing less gene members with respect to other herbaceous plant species, such as 45 members in *M. truncatula* [27,38], 54 in *O. sativa* [39], 35 in *Picea spp.* [22] and 44 in *L. usitatissimum* [40]. This suggests that some DIRs are missing from our analysis, possibly due to an incomplete transcriptome. According to our phylogenetic analysis, UdDIRs mainly clustered into subfamily-a and subfamily-b/d (Figure 2). It was demonstrated that members of subfamily-a have capabilities to form either (+)-PINO or (–)-PINO by stereoselective coupling of coniferyl alcohol, such as DIRs from *T. plicata* (TpDIR5) [41], *Podophyllum peltatum* [42], *F. x intermedia* [20] and *L. usitatissimum* (LuDIR1) [21]. Therefore, five UdDIRs that belong to subfamily-a (i.e., *UdDIR3/5/12/13/14*) are most likely involved in lignan formation (Figure 2). Moreover, RT-qPCR analyses of three subfamily-a *UdDIRs* showed spatial (i.e., different stem heights; TOP, MID and BOT internodes) and tissue-specific expression patterns (Figure 6A,B and Figure 7). Based on the gene expression level, *UdDIR12* could potentially contribute to the PINO biosynthesis in fibres, while *UdDIR13* could play an important role in forming PINO in young internodes.

Interestingly, a recent study showed that DIR22 from *Glycine max* (GmDIR22), a member of subfamily-b/d, is also involved in lignan biosynthesis [43]. In addition, a significant amino acid sequence homology was found between GmDIR22 and UdDIRs from subfamily-b/d (i.e., *UdDIR1/2/6/7/9/10/11*) (Table S4). Hence, it is plausible that these seven UdDIRs may partake in lignan biosynthesis. This may explain the predominant abundance of PINO in roots (Figure 5B), despite the low expression of subfamily-a members (*UdDIR5/12/13*) observed in the same tissue (Figure 6B). It is, therefore, tempting to speculate that *UdDIR1* and *UdDIR7*, two members of subfamily-b/d, could be implicated in PINO biosynthesis in roots, due to their high transcript abundance (Figure 6B). Further studies are needed to confirm the biochemical role of these *UdDIR*s.

Glycosylation via uridine diphosphate-glycosyltransferases (UGT) is a commonly occurring modification in lignan biosynthesis, as evidenced by the presence of diverse lignan glycosides in divergent plant species [44,45,46,47,48,49,50]. For instance, the most abundant lignan glycoside in sesame seeds is sesaminol triglucoside, while secoisolariciresinol diglucoside (SECO) is the major lignan in flaxseed. In this study, we observed the presence of PDG in all tissues of *U. dioica* clone 13, but, interestingly, with an opposite trend in relation to PINO content (Figure 5B). PDG is preferentially accumulated in young internodes (TOP and MID) and leaves, rather than older tissue (BOT) and roots, suggesting a possible role of PDG in the regulation of plant development. This is particularly relevant if one considers that, on the one hand, lignans were shown to affect plant growth [51,52,53] and, on the other hand, that fibre cells in the TOP and MID internodes are in the rapid elongation phase under a strict control involving gene regulatory network, reactive oxygen species and secondary metabolites [30].

Currently, the demand for PDG is rapidly increasing due to its pharmacological effects, such as antihypertension [54,55] and prevention of osteoporosis [56]. To date, Tu-chung (*Eucommia ulmoides* Oliv.) is the main source of PDG in nature; however, this tree grows very slow and the yield of PDG is also low [57]. Therefore, considerable efforts have been devoted to increasing the yield of PDG in vitro using a fungal strain [57,58,59]. Given the shorter growth cycle as compared to Tu-chung, nettle could be a good alternative source of PDG. In line with this, leaf extracts of *U. dioica* were demonstrated to be able to decrease both systolic and diastolic blood pressure [12], which could be partially attributed to the relatively high level of PDG in the leaves (Figure 5B). It would be very interesting to identify the UGTs that are involved in the glycosylation of PINO for metabolic engineering of PDG biosynthesis.

Most of PLRs catalyse two subsequent reductions from PINO to SECO via LARI [18,32], except for the ones from *A. thaliana* that have low or no affinity towards LARI [60]. Furthermore, it has been demonstrated that PLRs display enantiospecificity in both reduction steps, adding more complexity to the interpretation of the catalytic function of PLRs [18]. In our study, 3 *UdPLR*s were differentially expressed in various tissues (Figure 6C and Figure 7) and, therefore, could be responsible for the tissue-specific accumulation of lignans (perhaps with different enantiomeric composition) (Figure 5B and Figure 7). The phylogenetic analysis displayed that UdPLR1 clustered together with PLRs from other plant species that convert (+)-PINO to (+)-LARI and then to (–)-SECO, indicative of a similar enantiospecificity for UdPLR1. This result was further supported by the multiple sequence alignment of PLRs, showing that the amino acids associated with enantiospecificity were consistent between UdPLR1 and PLRs that convert (+)-PINO to (–)-SECO via (+)-LARI (Figure 4). Further investigations on the enantiospecificity and kinetic properties of each UdPLR will shed more light on this. Moreover, determining the enantiomeric configuration of nettle lignans via chiral HPLC will also advance our understanding of the biochemical role of UdPLRs.

We observed a higher expression level of *UdPLR1* and *UdPLR2* in TOP, MID, BOT-C and ROOT as compared to that in BOT-F and LEAF (Figure 6C), which is in line with the higher accumulation of LARI and SECO in these tissues (Figure 5B). It is worthwhile mentioning that the transcripts of *PLR*s were also found to be highly abundant in the inner stem tissue of flax, although no differences in the level of PINO and LARI were observed between inner and outer stem tissues (corresponding to BOT-C and BOT-F in this study, respectively) [61]. LARI and SECO were not detected in BOT-F (Figure 5B), notwithstanding *UdPLR*s were all expressed in BOT-F (Figure 6B). This discrepancy suggests a possible involvement of post-translational regulation mechanism. Concurrently with the development of prediction methods and bioinformatic tools [62], as part of future research, it is important to further identify the sites of post-translational modification for a better understanding of the molecular mechanisms involved in lignan biosynthesis. Post-translational regulation is known to play an important role in controlling key biosynthetic pathways of secondary metabolites, notably phenylpropanoids, through the regulation of the gateway enzyme phenylalanine ammonia lyase (PAL) [63].

As shown in a series of recent publications (see, e.g., [62,64]) demonstrating new findings or approaches, user-friendly and publicly accessible webservers will significantly enhance their impacts [65]), driving medicinal chemistry into an unprecedented revolution.

## 4. Material and Methods

### 4.1. Plant Material

The propagation, growth condition and sampling of *U. dioica* “clone 13” [66] were performed as described previously [30,67]. Briefly, the stem cuttings of plants were grown in growth chambers until about 70 cm tall under standard conditions, i.e., 25 °C 16 h light and 20 °C 8h dark. Different internodes were sampled along the stem. The TOP internode is located just below the apex. The middle internode (MID) shows a kink when tilting the plant (and may, therefore, include the snap point) and the BOT bottom internode (BOT) is the third internode underneath the MID. BOT internodes were peeled to separate core (BOT-C) and cortical tissues containing bast fibres (BOT-F). Four biological replicates (7 plants for each replicate) were collected for all stem samples, leaves (LEAF) and roots (ROOT).

### 4.2. Gene Identification and Phylogenetic Analysis

The predicted nettle *DIR*s and *PLR*s were identified via blasting the *Arabidopsis thaliana DIR*s and *PLR*s against the transcriptome of nettle “clone 13” [30] and nettle leaf database at China National GeneBank DataBase-oneKP (https://db.cngb.org/onekp/). The sequences of each gene were further examined via BLASTX analysis against the Viridiplantae database. The CDS and protein sequences of 14 DIRs and 3 PLRs are listed in Text S1.

Sequence alignments of DIRs and PLRs were carried out using CLUSTAL-Ω [68] and conserved residues were highlighted with Jalview [69].

The phylogenetic tree of both DIRs and PLRs was constructed. The alignment of full-length amino acid sequences constructed with CLUSTAL-Ω was subjected to phylogenetic analysis using maximum likelihood method via W-IQ-TREE with 1000 bootstraps [70]. The online program iTOL (https://itol.embl.de/) was used to visualise the tree.

A total of 218 DIRs protein sequences obtained from previous studies was used in the analysis [22,27,40,71]. These sequences are from the following plant species: *U. dioica (Ud)*, *Medicago truncatula* (Mt), *A. thaliana* (At), *Cannabis sativa* (Csa), *L. usitatissimum* (Lu), *Picea sitchensis* (P), *Oryza sativa* (Os), *Arachis hypogaea* (Ah), *Agrostis stolonifera* (As), *Forsythia x intermedia* (Fi), *Gossypium barbadense* (Gb), *Nicotiana benthamiana* (Nb), *Triticum aestivum* (Ta), *Hordeum vulgare* (Hv) and *Isatis indigotica* (Li). For PLRs, a total of 170 sequences from 79 plant species was used and obtained from previous studies [18,37]. The accession number of each protein is shown in Tables S1 and S2.

### 4.3. Lignan Extraction and Quantification

The extraction of lignans was performed on different organs and tissues of nettle plants with four biological replicates (7 plants for each replicate) and two technical replicates. The extraction procedure was adapted from [72]. Samples of 30 mg of ground lyophilised material were suspended in 1 mL of 300 mM NaOH (in 70% methanol, *v*/*v*) and placed in a thermomixer at 60 °C and 750 rpm for 1 h. To neutralise the extract, 20 µL of acetic acid were added after cooling to room temperature and 500 µL of supernatant were collected after centrifugation. The extract was then diluted 10 times with ultrapure water and filtered on a polytetrafluoroethylene membrane (cut-off 0.45 µm) and an injection volume of 10 µL was used for each analysis.

Quantitative analyses were carried out using Ultra-High Performance Liquid Chromatography (U-HPLC, 1290 Infinity II, Agilent, Waldbronn, Germany) coupled to high-resolution Quadrupole–Time of Flight (Q-ToF) mass spectrometry (X500R, Sciex, Darmstadt, Germany). The chromatographic separation was carried out on a BEH C18 column, 50 × 2.1 mm ID with the particle size of 1.8 µm (Waters, Milford, MA, USA). The flow rate of the mobile phase was kept constantly at 0.3 mL/min and the column oven was set at 40 °C. The mobile phases were LC-MS grade acetonitrile and 2.5 mM ammonium acetate in ultrapure water. The eluent gradient started with 5% of acetonitrile for 1 min, increased to 30% within 3 min, then to 90% within 1 min, kept at 90% for 2 min, returned to 5% within 1 min and equilibrated for 2 min. The mass spectrometer was operated in negative electrospray ionisation mode. The ion spray voltage was –4.5 kV and the source temperature 500 °C. Quantitative results were provided in High-Resolution Multiple Reaction Monitoring (MRM-HR) mode. Results were confirmed by a second MRM-HR transition and by the ToF-MS signal. Six standard lignans (Sigma-Aldrich, Darmstadt, Germany) were used, i.e., pinoresinol (PINO), pinoresinol diglucoside (PDG), lariciresinol (LARI), secoisolariciresinol (SECO), secoisolariciresinol diglucoside (SDG) and matairesinol (MATA). The amount of each lignan was calculated against an external calibration curve obtained by different standard concentrations ranging from 1–200 ng/mL. A one-way ANOVA with Tukey’s post-hoc test was carried out to determine the significant differences among groups using SPSS 13.0 (SPSS Inc., Chicago, IL, USA).

### 4.4. Total RNA Extraction, cDNA Synthesis and Quantitative Real-Time PCR (RT-qPCR)

Total RNA extraction, cDNA synthesis and RT-PCR were carried out as previously reported [64]. Primers were designed using “Primer3Plus” [73]. Seven serial dilutions of cDNA (12.5, 2.5, 0.5, 0.1, 0.02, 0.004, 0.0008 ng/µL) were used to calculate the primer efficiency. Primer sequences and their primer efficiency are provided in Table 1. Five reference genes published previously were used in this study (i.e., *RAN*, *EF2,* tubulin and *eTIF4E*) [64] and the normalisation of data was performed using *RAN* and *EF2,* which were identified as the most stable genes by geNorm^TM^, as implemented in the qbase+ software (Biogazelle, Zwijnaarde, Belgium). The log2 transformed data were used for statistics using a one-way ANOVA followed by a Tukey’s post-hoc test (SPSS 13.0, SPSS Inc.). Hierarchical clustering of gene expression data was carried out using Cluster 3.0 [74] with Pearson correlation and complete linkage and the heat map was visualised with Java TreeView.

## Figures and Tables

**Table 1:** Details of the identified dirigent proteins (*DIR*s) and pinoresinol-lariciresinol reductase (*PLR*s) in *U. dioica* with proposed nomenclature, transcript ID and open reading frame (ORF) length, primer sequences for RT-qPCR analysis and amplification efficiencies.

Nomenclature	Transcript ID	ORF Length	Forward Primer (5′→3′)	Reverse Primer (5′→3′)	Efficiency (%)	R2
UdDIR1 *	contig_12966	191	TCTCATGGTCCTCAACTACGTC	TTCCGTCCCAATATGCTGAG	99.474	0.99
UdDIR2 *	contig_14063	192	TCTCAAGCTCACACGCAAAC	AGAGCTTTTCTCTGCGGAGTC	91.93	0.997
UdDIR3	contig_22204	186				
UdDIR4 ^#^	contig_23037	103				
UdDIR5 *	contig_24527	201	GTCATCAAGCCATGCAAGAG	TCTTGAGGTTGTGACCGTTG	103.679	0.99
UdDIR6	contig_24857	178				
UdDIR7 *^,#^	contig_28042	143	ACGTAGTTCTGGACCATGAGG	ATTATCGACGACCCGTTGAC	90.92	0.997
UdDIR8 ^#^	contig_28614	135				
UdDIR9 *	contig_28699	191	GGCCAAATCAAAGGAGACAG	ACCCCGTTTTCGATAAGGTC	94.912	0.988
UdDIR10 ^#^	contig_32790	85				
UdDIR11 *^#^	contig_34554	160	GGGAAACCTTCATGATCGAC	TGACCATGAGTAGGGCAATG	96.397	0.998
UdDIR12 *	contig_34733	189	GGGCACTTTGAACGTAATGG	TCCTTGGTAAGTGTCGGTCTG	94.462	0.998
UdDIR13 *^,#^	contig_34949	183	TCACAGCGTCGAAAGACAAC	TCACGCGTCATCTTGTCATC	94.294	0.995
UdDIR14	contig_7375	203				
UdPLR1 *^,#^	contig_26577	308	CGCCTCTTTCGAAGACAAAG	AGAAGGATGTGATGGGTTCG	94.212	0.995
UdPLR2 *	contig_628	312	CTCGTCGAAGGTTCGTTTTC	CCGGCTTCTTTAATGGCTTC	99.362	0.998
UdPLR3 *	contig_10583	309	CGAAAATGGAGGAGCAGAAG	AAGGTGGGATGAGATGATCG	101.629	0.995

* Genes selected for the RT-qPCR analysis; ^#^ Genes with an incomplete ORF.

## Supplementary Materials

The following are available online, Text S1: CDS and protein sequences of nettle DIRs and PLRs, Figure S1: phylogenetic analysis of UdPLRs and PLRs that were characterised for the enantiospecificity, Table S1: list of the dirigent protein (DIR) accession numbers and amino acid length from different plant species used in this study, Table S2: list of the pinoresinol-lariciresinol reductase (PLR) protein accession numbers and amino acid length from different plant species used in this study, Table S3: the RPKM value of *UdDIR*s and *UdPLR*s in different tissues obtained from our previous transcriptomic analysis, Table S4: amino acid sequence homology between DIR22 from Glycine max (accession number: ADX66343.1) and UdDIRs from subfamily-b/d.

## References

[1] Akiyama K., Yamauchi S., Nakato T., Maruyama M., Sugahara T., Kishida T. (2007). Antifungal activity of tetra-substituted tetrahydrofuran lignan,(−)-virgatusin and its structure-activity relationship. Biosci. Biotechnol. Biochem..

[2] Gang D.R., Kasahara H., Xia Z.-Q., Vander Mijnsbrugge K., Bauw G., Boerjan W., Van Montagu M., Davin L.B., Lewis N.G. (1999). Evolution of plant defense mechanisms relationships of phenylcoumaran benzylic ether reductases to pinoresinol-lariciresinol and isoflavone reductases. J. Biol. Chem..

[3] Naoumkina M.A., Zhao Q., Gallego-Giraldo L., Dai X., Zhao P.X., Dixon R.A. (2010). Genome-wide analysis of phenylpropanoid defence pathways. Mol. Plant Pathol..

[4] Zhang J., Chen J., Liang Z., Zhao C. (2014). New lignans and their biological activities. Chem. Biodiver..

[5] Bartosz T., Irene T. (2016). Polyphenols encapsulation–application of innovation technologies to improve stability of natural products. Phys. Sci. Rev..

[6] Francišković M., Gonzalez-Pérez R., Orčić D., Sánchez de Medina F., Martínez-Augustin O., Svirčev E., Simin N., Mimica-Dukić N. (2017). Chemical Composition and Immuno-Modulatory Effects of *Urtica dioica* L.(Stinging Nettle) Extracts. Phytother. Res..

[7] Schöttner M., Ganßer D., Spiteller G. (1997). Lignans from the roots of *Urtica dioica* and their metabolites bind to human sex hormone binding globulin (SHBG). Planta Med..

[8] Asgarpanah J., Mohajerani R. (2012). Phytochemistry and pharmacologic properties of *Urtica dioica* L.. J. Med. Plant Res..

[9] Chrubasik S., Enderlein W., Bauer R., Grabner W. (1997). Evidence for antirheumatic effectiveness of Herba *Urticae dioicae* in acute arthritis: A pilot study. Phytomedicine.

[10] Legssyer A., Ziyyat A., Mekhfi H., Bnouham M., Tahri A., Serhrouchni M., Hoerter J., Fischmeister R. (2002). Cardiovascular effects of *Urtica dioica* L. in isolated rat heart and aorta. Phytother. Res..

[11] Roschek B., Fink R.C., McMichael M., Alberte R.S. (2009). Nettle extract (*Urtica dioica*) affects key receptors and enzymes associated with allergic rhinitis. Phytother. Res..

[12] Vajic U.-J., Grujic-Milanovic J., Miloradovic Z., Jovovic D., Ivanov M., Karanovic D., Savikin K., Bugarski B., Mihailovic-Stanojevic N. (2018). *Urtica dioica* L. leaf extract modulates blood pressure and oxidative stress in spontaneously hypertensive rats. Phytomedicine.

[13] Tahri A., Yamani S., Legssyer A., Aziz M., Mekhfi H., Bnouham M., Ziyyat A. (2000). Acute diuretic, natriuretic and hypotensive effects of a continuous perfusion of aqueous extract of *Urtica dioica* in the rat. J. Ethnopharmacol..

[14] Schöttner M., Reiner J., Tayman F.S. (1997). (+)-neo-olivil from roots of *Urtica dioica*. Phytochemistry.

[15] Kraus R., Spiteller G. (1990). Phenolic compounds from roots of *Urtica dioica*. Phytochemistry.

[16] Teponno R.B., Kusari S., Spiteller M. (2016). Recent advances in research on lignans and neolignans. Nat. Prod. Rep..

[17] Ward R.S. (1993). Lignans neolignans and related compounds. Nat. Prod. Rep..

[18] Markulin L., Corbin C., Renouard S., Drouet S., Gutierrez L., Mateljak I., Auguin D., Hano C., Fuss E., Lainé E. (2019). Pinoresinol–lariciresinol reductases, key to the lignan synthesis in plants. Planta.

[19] Umezawa T. (2003). Diversity in lignan biosynthesis. Phytochem. Rev..

[20] Davin L.B., Wang H.-B., Crowell A.L., Bedgar D.L., Martin D.M., Sarkanen S., Lewis N.G. (1997). Stereoselective bimolecular phenoxy radical coupling by an auxiliary (dirigent) protein without an active center. Science.

[21] Dalisay D.S., Kim K.W., Lee C., Yang H., Rúbel O., Bowen B.P., Davin L.B., Lewis N.G. (2015). Dirigent protein-mediated lignan and cyanogenic glucoside formation in flax seed: Integrated omics and MALDI mass spectrometry imaging. J. Nat. Prod..

[22] Ralph S.G., Jancsik S., Bohlmann J. (2007). Dirigent proteins in conifer defense II: Extended gene discovery, phylogeny and constitutive and stress-induced gene expression in spruce (*Picea* spp.). Phytochemistry.

[23] Vassão D.G., Kim K.-W., Davin L.B., Lewis N.G. (2010). Lignans (neolignans) and allyl/propenyl phenols: Biogenesis, structural biology and biological/human health considerations. Chem. Biol..

[24] Hosmani P.S., Kamiya T., Danku J., Naseer S., Geldner N., Guerinot M.L., Salt D.E. (2013). Dirigent domain-containing protein is part of the machinery required for formation of the lignin-based Casparian strip in the root. Proc. Natl. Acad. Sci. USA.

[25] Davin L.B., Jourdes M., Patten A.M., Kim K.-W., Vassão D.G., Lewis N.G. (2008). Dissection of lignin macromolecular configuration and assembly: Comparison to related biochemical processes in allyl/propenyl phenol and lignan biosynthesis. Nat. Prod. Rep..

[26] Jin-long G., Li-ping X., Jing-ping F., Ya-chun S., Hua-ying F., You-xiong Q., Jing-sheng X. (2012). A novel dirigent protein gene with highly stem-specific expression from sugarcane, response to drought, salt and oxidative stresses. Plant Cell Rep..

[27] Behr M., Legay S., Hausman J.-F., Guerriero G. (2015). Analysis of cell wall-related genes in organs of *Medicago sativa* L. under different abiotic stresses. Inter. J. Mol. Sci..

[28] Fujita M., Gang D.R., Davin L.B., Lewis N.G. (1999). Recombinant pinoresinol-lariciresinol reductases from western red cedar (*Thuja plicata*) catalyze opposite enantiospecific conversions. J. Biol. Chem..

[29] Hemmati S., von Heimendahl C.B., Klaes M., Alfermann A.W., Schmidt T.J., Fuss E. (2010). Pinoresinol-lariciresinol reductases with opposite enantiospecificity determine the enantiomeric composition of lignans in the different organs of *Linum usitatissimum* L.. Planta Med..

[30] Xu X., Backes A., Legay S., Berni R., Faleri C., Gatti E., Hausman J.F., Cai G., Guerriero G. (2019). Cell wall composition and transcriptomics in stem tissues of stinging nettle (*Urtica dioica* L.): Spotlight on a neglected fibre crop. Plant Direct.

[31] Min T., Kasahara H., Bedgar D.L., Youn B., Lawrence P.K., Gang D.R., Halls S.C., Park H., Hilsenbeck J.L., Davin L.B. (2003). Crystal structures of pinoresinol-lariciresinol and phenylcoumaran benzylic ether reductases and their relationship to isoflavone reductases. J. Biol. Chem..

[32] von Heimendahl C.B., Schäfer K.M., Eklund P., Sjöholm R., Schmidt T.J., Fuss E. (2005). Pinoresinol–lariciresinol reductases with different stereospecificity from *Linum album* and *Linum usitatissimum*. Phytochemistry.

[33] Bayindir U., Alfermann A.W., Fuss E. (2008). Hinokinin biosynthesis in *Linum corymbulosum* Reichenb. Plant J..

[34] Dinkova-Kostova A.T., Gang D.R., Davin L.B., Bedgar D.L., Chu A., Lewis N.G. (1996). (+)-Pinoresinol/(+)-lariciresinol reductase from *Forsythia intermedia*. Protein purification, cDNA cloning, heterologous expression and comparison to isoflavone reductase. J. Biol. Chem..

[35] Wankhede D.P., Biswas D.K., Rajkumar S., Sinha A.K. (2013). Expressed sequence tags and molecular cloning and characterization of gene encoding pinoresinol/lariciresinol reductase from *Podophyllum hexandrum*. Protoplasma.

[36] Lau W., Sattely E.S. (2015). Six enzymes from mayapple that complete the biosynthetic pathway to the etoposide aglycone. Science.

[37] Wu Y., Xing D., Ma G., Dai X., Gao L., Xia T. (2019). A variable loop involved in the substrate selectivity of pinoresinol/lariciresinol reductase from *Camellia sinensis*. Phytochemistry.

[38] Song M., Peng X. (2019). Genome-Wide Identification and Characterization of DIR Genes in *Medicago truncatula*. Biochem. Genet..

[39] Liao Y., Liu S., Jiang Y., Hu C., Zhang X., Cao X., Xu Z., Gao X., Li L., Zhu J. (2017). Genome-wide analysis and environmental response profiling of dirigent family genes in rice (*Oryza sativa*). Genes Genom..

[40] Corbin C., Drouet S., Markulin L., Auguin D., Lainé É., Davin L.B., Cort J.R., Lewis N.G., Hano C. (2018). A genome-wide analysis of the flax (*Linum usitatissimum* L.) dirigent protein family: From gene identification and evolution to differential regulation. Plant Mol. Biol..

[41] Kim M.K., Jeon J.-H., Fujita M., Davin L.B., Lewis N.G. (2002). The western red cedar (*Thuja plicata*) 8-8′ DIRIGENT family displays diverse expression patterns and conserved monolignol coupling specificity. Plant Mol. Biol..

[42] Xia Z.-Q., Costa M.A., Proctor J., Davin L.B., Lewis N.G. (2000). Dirigent-mediated podophyllotoxin biosynthesis in *Linum flavum* and *Podophyllum peltatum*. Phytochemistry.

[43] Li N., Zhao M., Liu T., Dong L., Cheng Q., Wu J., Wang L., Chen X., Zhang C., Lu W. (2017). A novel soybean dirigent gene *GmDIR22* contributes to promotion of lignan biosynthesis and enhances resistance to *Phytophthora sojae*. Front. Plant Sci..

[44] Ayres D.C., Loike J.D. (1990). Lignans: Chemical, Biological and Clinical Properties.

[45] Palter R., Lundin R., Haddon W. (1972). A cathartic lignan glycoside isolated from *Cart*. Tinctorus. Phytochem..

[46] Katsuzaki H., Kawasumi M., Kawakishi S., Osawa T. (1992). Structure of novel antioxidative lignan glucosides isolated from sesame seed. Biosci. Biotechnol. Biochem..

[47] Katsuzaki H., Kawakishi S., Osawa T. (1994). Sesaminol glucosides in sesame seeds. Phytochemistry.

[48] Moazzami A.A., Andersson R.E., Kamal-Eldin A. (2006). HPLC analysis of sesaminol glucosides in sesame seeds. J. Agric. Food Chem..

[49] Moazzami A.A., Andersson R.E., Kamal-Eldin A. (2006). Characterization and analysis of sesamolinol diglucoside in sesame seeds. Biosci. Biotechnol. Biochem..

[50] Noguchi A., Fukui Y., Iuchi-Okada A., Kakutani S., Satake H., Iwashita T., Nakao M., Umezawa T., Ono E. (2008). Sequential glucosylation of a furofuran lignan,(+)-sesaminol, by *Sesamum indicum* UGT71A9 and UGT94D1 glucosyltransferases. Plant J..

[51] Yamauchi S., Ichikawa H., Nishiwaki H., Shuto Y. (2015). Evaluation of plant growth regulatory activity of furofuran lignan bearing a 7, 9′: 7′, 9-Diepoxy structure using optically pure (+)-and (−)-Enantiomers. J. Agric. Food Chem..

[52] Cutillo F., D’Abrosca B., DellaGreca M., Fiorentino A., Zarrelli A. (2003). Lignans and neolignans from *Brassica fruticulosa*: Effects on seed germination and plant growth. J. Agric. Food Chem..

[53] Yamauchi S., Kumamoto M., Ochi Y., Nishiwaki H., Shuto Y. (2013). Structure–Plant Growth Inhibitory Activity Relationship of Lariciresinol. J. Agric. Food Chem..

[54] Sih C.J., Ravikumar P., Huang F.-C., Buckner C., Whitlock H. (1976). Isolation and synthesis of pinoresinol diglucoside, a major antihypertensive principle of Tu-Chung (*Eucommia ulmoides*, Oliver). J. Am. Chem. Soc..

[55] Luo L.-F., Wu W.-H., Zhou Y.-J., Yan J., Yang G.-P., Ouyang D.-S. (2010). Antihypertensive effect of *Eucommia ulmoides* Oliv. extracts in spontaneously hypertensive rats. J. Ethnopharmacol..

[56] Saleem M., Kim H.J., Ali M.S., Lee Y.S. (2005). An update on bioactive plant lignans. Nat. Prod. Rep..

[57] Zhang Y., Shi J., Liu L., Gao Z., Che J., Shao D., Liu Y. (2015). Bioconversion of pinoresinol diglucoside and pinoresinol from substrates in the phenylpropanoid pathway by resting cells of *Phomopsis sp.* XP-8. PLoS ONE.

[58] Zhang Y., Shi J., Gao Z., Yangwu R., Jiang H., Che J., Liu Y. (2015). Production of pinoresinol diglucoside, pinoresinol monoglucoside and pinoresinol by *Phomopsis sp.* XP-8 using mung bean and its major components. Appl. Microbiol. Biotechnol..

[59] Zhang Y., Shi J., Gao Z., Che J., Shao D., Liu Y. (2016). Comparison of pinoresinol diglucoside production by *Phomopsis sp.* XP-8 in different media and the characterisation and product profiles of the cultivation in mung bean. J. Sci. Food Agric..

[60] Nakatsubo T., Mizutani M., Suzuki S., Hattori T., Umezawa T. (2008). Characterization of *Arabidopsis thaliana* pinoresinol reductase, a new type of enzyme involved in lignan biosynthesis. J. Biol. Chem..

[61] Huis R., Morreel K., Fliniaux O., Lucau-Danila A., Fénart S., Grec S., Neutelings G., Chabbert B., Mesnard F., Boerjan W. (2012). Natural hypolignification is associated with extensive oligolignol accumulation in flax stems. Plant Physiol..

[62] Chou K.C. (2019). Progresses in predicting post-translational modification. Int. J. Pept. Res. Ther..

[63] Zhang X., Liu C.J. (2015). Multifaceted regulations of gateway enzyme phenylalanine ammonia-lyase in the biosynthesis of phenylpropanoids. Mol. Plant.

[64] Liu B., Wang S., Long R., Chou K.C. (2017). iRSpot-EL: Identify recombination spots with an ensemble learning approach. Bioinformatics.

[65] Chou K.C. (2015). Impacts of bioinformatics to medicinal chemistry. Med. Chem..

[66] Bacci L., Baronti S., Predieri S., Di Virgilio N. (2009). Fiber yield and quality of fiber nettle (*Urtica dioica* L.) cultivated in Italy. Ind. Crop. Prod..

[67] Backes A., Behr M., Xu X., Gatti E., Legay S., Predieri S., Hausman J.-F., Deyholos M.K., Cai G., Guerriero G. (2018). Sucrose synthase gene expression analysis in the fibre nettle (*Urtica dioica* L.) cultivar “clone 13”. Ind. Crop. Prod..

[68] McWilliam H., Li W., Uludag M., Squizzato S., Park Y.M., Buso N., Cowley A.P., Lopez R. (2013). Analysis tool web services from the EMBL-EBI. Nucleic Acids Res..

[69] Waterhouse A.M., Procter J.B., Martin D.M., Clamp M., Barton G.J. (2009). Jalview Version 2—a multiple sequence alignment editor and analysis workbench. Bioinformatics.

[70] Trifinopoulos J., Nguyen L.-T., von Haeseler A., Minh B.Q. (2016). W-IQ-TREE: A fast online phylogenetic tool for maximum likelihood analysis. Nucleic Acids Res..

[71] Behr M., Sergeant K., Leclercq C.C., Planchon S., Guignard C., Lenouvel A., Renaut J., Hausman J.-F., Lutts S., Guerriero G. (2018). Insights into the molecular regulation of monolignol-derived product biosynthesis in the growing hemp hypocotyl. BMC Plant Biol..

[72] Milder I.E.J., Arts I.C.W., Venema D.P., Lasaroms J.J.P., Wähälä K., Hollman P.C.H. (2004). Optimization of a Liquid Chromatography−Tandem Mass Spectrometry Method for Quantification of the Plant Lignans Secoisolariciresinol, Matairesinol, Lariciresinol and Pinoresinol in Foods. J. Agric. Food Chem..

[73] Untergasser A., Nijveen H., Rao X., Bisseling T., Geurts R., Leunissen J.A. (2007). Primer3Plus, an enhanced web interface to Primer3. Nucleic Acids Res..

[74] De Hoon M.J., Imoto S., Nolan J., Miyano S. (2004). Open source clustering software. Bioinformatics.

