# Supplementary material for: Insights into Lignan Composition and Biosynthesis in Stinging Nettle (Urtica dioica L.)
Source: Molecules. 2019 Oct 26;24(21):3863. doi: 10.3390/molecules24213863 (PMC6864805; doi:10.3390/molecules24213863)
Supplement: Supplementary file 1 [file molecules-24-03863-s001.zip › Text S1.docx]

**Text S1. CDS and protein sequences of nettle DIRs and PLRs**

>UdDIR1_contig-12966_CDS

ATGATCATGAAAACCCTCATCATCAAGGCTATTACCATTTTGTCCCTCCTAGCCCTAGCAAGTTCTAACGACCAAAAATACCACAAGAGCGTGTCCCCAAAAGCCCTAGGGCTCCACAAGAGAGAGAAGCTCACCCACATCCACTTCTACTTCCACGACGTGGTGGCCGGAAAAAACGCCACGGTGGTCCACGTGGCGGGCCCGCCGGTGGGGCTCGGGGGCCCGAGGGAGAGCTTCGGGTCCGTGGTGGTGATGGACGACCCGTTGACCGTGGGGCCCGACCCGGGGTCGAAGCGGATCGGGAGCGCCCAAGGGATCTACTCCTCGGCGTCTCAGACCGAGCTAGGGTTTCTCATGGTCCTCAACTACGTCTTCACCGACGGAAAATTCAAGGGAAGTACTCTCAGCATATTGGGACGGAACGCGATTCTCGATGAGGTCAGAGAAATGCCCGTCGTCGGAGGCACCGGGGTTTTCAGGTTCGCCACAGGGTTCGCTCAGGCCAGGACTCACGTGTTCAACACCACCACTCTTGACGCCATTGTTGAGTACAACGTCTATGTCCTCCATTAT

>UdDIR2_contig-14063_CDS

ATGGTGAGAAGAACTCAAAAAACCCTCCTCTCATCTCTCATAACAATCCTATTCCTCTTCTCAATCTCAAGCTCACACGCAAACAAATACCACAAAACCCTAAACCCAAGAACCCTAGGACTCCGCAGAGAAAAGCTCTCCCACCTCAAATTCTACTTCCACGACGTCGTGAGCGGCAAATCCCCGACGGTGGTCCGAGTCGCCTCATCCCCGACGACCAACACGTCACCGACGAGCTTCGGATTCGTCGCCGTGATGGACGACCCGTTGACCCTCGGACCGGAAATCAACTCGACACGTGTCGGAAGCGCTCAGGGGATCTACGCCTCGGCGTCTCAGACGGAGCTGGGACTCTTGATGGTCCACACGTACGCATTCACCGCCGGaAAATATAACGGAAGCTCTCTCAGTATTCTGGGACGAAACGCCGTGTTCAACGCCGTCAGGGAGCTTCCGATCGTCGGAGGGACGGGGTTGTTCCGGTTCGCGCGTGGCTACGCGCTCGCCAAGACGCACGTGTTCGACGGCAAGTCTGGGGACGCTGTTGTTAAGTACAATGTCTATGTGTTGCATTAT

>UdDIR3_contig-22204-CDS

ATGAGAACCATTTCATCAAATTGGGGTTTCATTTTCCTTTCCCTCTTAGCAATAATAGCTCTATTTTATTCCCAACCGATTTCCGCGGCCAATAAGAAACGCCCTATATCGCCATGCAAGCGTTTCGTCGTCCACTACCACGACGTCATGTTCAACGGCACAAACTCGGTCAACGCCACGGCAGCGGTGGTGGTCAACGATACAGCGCTCAGTCAAACCAAGTTCGGGAAATTCGTGGTTTTCGACGACAAAATGACGTACGACGAAAACCTAAGCTCCCCCGAGGCCGCTAGGGCTCAAGGCTTCTACATCTACGACATCAAGGAAAGTTATGGCGCGTGGTTCGCTTTCTCTCTCATATTTAATTCGACCGAGCACAAGGGCACTTTGAACCTCCTCGGAGCAGATTTGATGGCGGAAAAGACCAGGGATCTTTCGGTGGTCGGCGGCACGGGGGATTTCTTCATGGCGCGAGGGATCGCCTCTGTTGAGACCGATTCTTTTGAGGGTTTTAGGTATTTTCGCCTCAAAATGGACATTAAGTTGTATGAGTGCTAT

>UdDIR4_contig-23037_CDS

ATGGTGCAAAAGATCTCTGAAAATTCTAGGATTTCGCTCTGCATTTCCGTGGCTTTGGCCGTGTTGGTGGTCGTTCTCTTGGCCGTCTTCACGCCCAAACTCCACAACTCGTCGCCCAGCCAGAACGCTGCAGAGTGGCTCGACCTCTCGCTCTACATCCAAGAGCCGCCCCGCGGGGCCCACGGGGGGGATCCCNNGGCGGCGGCGGCAGCGGTGGCGGCTGATCAGGCGGACGCCGGAGCGCTGGTCTTCCACCGCACCCTCACGGAGGGGCCGGAGAACACGTCGAGGATCGTGGGGAAAGCTCAGGG

>UdDIR5_contig-24527_CDS

ATGGCGTCTAATTCAATAATTTCGATTTCGCTGCTCTTCCTCGTCCTCGTCCTCTTGATCTCCCCGGGCTCGAACGCCACGCCGAATAAGAAATCGACACGTCACCGCGGCCGCCACGGAATCAACGTCATCAAGCCATGCAAGAGAATGGTGTTCTATTTCCACGACATCATATACAACGGTCACAACCTCAAGAACGCCACGTCATCGATAGTCGGCGAGCCGGCTTGGGGGAACAAGACGATCCTGGCGAGCCAAAACCACTTTGGCAACGTGGTCGTTTTCGACGACCCGATAACCCTAGACAACGATCTCCACTCAGCTCCGGTCGGAAGGGCTCAAGGGTTTTACTTGTACGACAAGCGAGACGTTTTCACGTCGTGGCTAGGGTTCTCTTTCGTGTTCGATTCCGAGGAGCACAAGGGAAGCATAAACTTTGCCGGGGCTGATCCGCTCATGAACAAGACCAGGGACATTTCGGTCATTGGCGGGACCGGAGATTTCTTCATGGCTAGAGGGATCGCCACGTTGATGACTGACGCGTTCGAAGGTGAAGTTTATTTCAGGCTTCGCGTTGATATCAAGCTCTACGAGTGTTGGGTT

>UdDIR6_contig-24857_CDS

ATGGCCAAAC TGATCATTAG CTTCACTTCC ATGATCATGA TCACAATGAT GATCATCTCTTCCACGAACG CAGAATTCGC CAGGAAAGAA GAGGGAGTGA CACATCTAGA GTTCTACTTC CACGACACGT TGAGCGGGGA AGCCCCGAGC GCCGTAAGGG TGGCCGATGC CGGAGAAATT ACGAACAAGT CCCTGACCGT CTTCGGGGCG GTCCTCATGG CCGACGACCC GCTGACCGAG ACCCCCGACC CGGGATCCAA GCTGGTGGGC CGGGCCCAAG GGCTGTACGG GTCGGCGGGC CAGCACGAGC TGGCTCTCAT CATGGCCATG AACTTCGGGT TCATCGAAGG AGAGTACAAC GGGAGCTCGA TCAGCATCCT CGGCAAGAAC TCGGCAATGA GTCCCGTCCG AGAAATGGCG GTCGTCGGCG GGACCGGCGT GTTCCGGATG GCGCGTGGCT ACGCAATCGC TCACACTCAT TGGTTCAACC TCCTCGGCGA TGCTATAGTT GGATACAATG TCACTATTGT

>UdDIR7_contig-28042_CDS

TTCTACTTCCACGACGTCGTGAGCGGCAAAAATCCGACCGCCGTGCGAGTCGCCGAGGCTCCGACGACCAACACCTCGGCCACGCTGTTCGGAGCGATCGTCATTATCGACGACCCGTTGACTCTCGGACCGGACCCGAAATCCCCGAGGCTCGGGAGCGCCCAGGGGTTCTACTCCACGGTGTCCCAGTCCGACGTGGTGCTCCTCATGGTCCAGAACTACGTCTTCACTGCCGGGAAGTTCAACGGAAGCACTCTCAGCGTGCTCGGCCGGAACGCGCCGTTCGCCGCCGTGAGGGAACTTCCGGTGGTCGGAGGGACCGGCCGGTTCAGGTTCGCCAGAGGGTTCGCTCTGGCCAAGACCCATTTCTACAACGCCACTACCGGTGACGCAATTGTCCAGTACAATGTCTACGTCAACCATTATTAT

>UdDIR8_contig-28614_CDS

ATGGCCAAACTGATCATTAGCTTCACTTCCATGATCATGATCACAATGATGATCATCTCTTCCACGAACGCAGAATTCGCCAGGAAAGAAGAGGGAGTGACACATCTAGAGTTCTACTTCCACGACACGTTGAGCGGGGAAGCCCCGAGCGCCGTAAGGGTGGCCGATGCCGGAGAAATTACGAACAAGTCCCTGACCGTCTTCGGGGCGGTCCTCATGGCCGACGACCCGCTGACCGAGACCCCCGACCCGGGATCCAAGCTGGTGGGCCGGGCCCAAGGGCTGTACGGGTCGGCGGGCCAGCACGAGCTGGCTCTCATCATGGCCATGAACTTCGGGTTCATCGAAGGAGAGTACAACGGGAGCTCGATCAGCATCCTCGGCAAGAACTCGGCAATGAGTCCCGTCCGAGAAATGGCGGTCGTCGGCGGGACCGGCGTGTTCCGGATGGCGCGTGGCTACGCAATCGCTCACACTCATTGGTTCAACCTCCTCGGCGATGCTATAGTTGGATACAATGTCACTATTGTTCAC

>UdDIR9_contig-28699_CDS

ATGGCAAAAAAACTGACCGTCACAACAATCCTCTCGCTAGCTCTACTACTCTCAGTCTCATCAGTGCTATCATCATCAACGACAAAGTCAAAGTCACGGGAAAAGCTGATCATGAAAATGGGTTTGAAGAAGCAAAAGCTGAGCCACTTCAAGTTCTTCTGGCACGACAAGTACAGCGGCAAGAACCCCACCGCCGTCACCATCGTCGCGCCCCCCAACGACACGTCGTTTTTCGGGAGCCTGACGATGATCGACGACGCTCTGACGGAGCGGGCCGACCCGAATTCGAGGCTTCTGGGGCGGGCCCAGGGACTGTACGGGTCGGCCTCGCGTGGCCAAGTGGCGCTGATCATGGCCATGAACTTCCACTTCGTCCAAGGCAAGTACAACGGTAGCGGAATTACCATTTTGGGTCGCAACCCCGTTTTCGATAAGGTCCGTGAAATGCCCGTGATCGCCGGGACCGGACTTTTTCGCTTCGCACGTGGGTTCGCACGTGCGAGCACTGTCTCCTTTGATTTGGCCACCGGGGACGCCGTGGTCCAGTACAATGTCTACGTGCTTCATTATGGC

>UdDIR10_contig-32790_CDS

GGATCTACGCCTCGGCGTCTCAGAGCGACCTATCGCTCCTCGAGATCTTCAACTACGTGTTCACGGACGGCGAGTTCAAGGGAAGCTCGCTGAGCCTCTTGGGACGGAACGCGATCCTCGAGGAGGTCAGAGAAATGCCTATCCTCGGAGGGACCGGGGTTTTCAGGTTCTGCAGAGGCTATGCTCTTCTCAAGACTAACATGTTCAACACCACCAGTGGTGACGCCATTGTTGAGTACGACGTCTATGTGCTTCAT

>UdDIR11_contig-34554_CDS

ATGACCATTCTCTTCATCCTCAGCCTGATCTCAACCTCATTTTTCATCTCTACCAATGGAGGGTTTTTGAGCGAATCCAAGGCGCTTAACAAGACCGAAAAACTAAGCCACTTTCACTTCTACTTCCACGACGTTCTGAGCGGGAAAAACCCGTCCGCAATCAAAATAATCAACCCGCCGATCCCAAAGGCGGGCTTGGGCTTGTTCGGGGAAACCTTCATGATCGACGATGCTTTGACCGTGGGACCAGACCCGAAATCGAAGACCGTGGGAAGAGCTCAAGGCTTGTACTCGATAGCGACGCAAAAAAGCATTGCCCTACTCATGGTCATGAACTTCGCGTTTGTGGACGGAAAGTACAACGGAAGTTGCGTTAGTGTCCTCGGAAGAAACTCGGTTTTCGACGACGTTAGAGAGCTTCCGATAGTCGGAGGGACCGGATCGTTTAGGTTTGCTCGCGGATACGCTTTGGCTCACACG

>UdDIR12_contig-34733_CDS

AAACCCTCATCAAGTGTCTTTCTCTCCATCCTTTTGTTCTTGGTGATCGTCTTAATAACATCCGAATCTTGCTCCGCAAGTAATATTAATAACAAGAAACCCTACATCAAAGAGAGCTCGCCGTGCAAGCGATTCGTGCTCTATTACCACGACGTCCTCTACAACGGCACGGACTTGGCCAACGCCACGTCAGCAACGATCGCGAACAAGACCGCTCTCGGGCCTTACAATTTCGGAAAGTTTGTCGTTTTCAACGACAAGTTGACGCGGGACAACAAGCTTCGCTCGCCCGAGGTCGCGAGAGCGCACGGGTTCTTCTTCTACGACATGAAGACCACGTACAACGCGTGGTTCGCGTTCACTTTGGTCTTTAACTCGAGCGATTACAAGGGCACTTTGAACGTAATGGGCGCGGATATGATGTCGGAGAAGACTAGGGACCTTTCGGTGGTCGGAGGCACGGGAGATTTCTTCATGACTCGAGGTATCGTCACGATTCAGACCGACACTTACCAAGGAGACTTTTATTTTAGGCTTAAGATGGATATTAAGCTCTACGAGTGCTAC

>UdDIR13_contig-34949_CDS

AACATGAGAA CATCAAAACC CTCATCAAGT TTTTTCTTTC CCATCATTCT AATCTTCGTGATCCTCCTAA TAACATCCGA ATCTTGTTTC GCAGAAAAGA AACCCTACAT CAAAGAGAGCTCTCCGTGCA AGCGATTCGT GCTCTATTAC CACGACATCC ACTTCAACGG CACTAACGCGGCCAACGCAA CCTCAGTCAC AGCGTCGAAA GACAACCCTT TCGGCGGGCC CTACTTTTTCGGGAGACTTG TCGTTTTCGA TGACAAGATG ACGCGTGACA ACAAGCCCGA TTCTCCGAGCGTGGCTAGGG CTCAGGGATT TTACTTCTAC GACATGAAGA CCAAGTACAA CGCGTGGTTCGCGTACACTC TCGTCTTCGA TTCGAGCGAG CACAAGGGCT CTTTGAACAT CATGGGCGCTAATACGATTT CGGAGAAGAC TAGGGATCTT TCGGTGGTCG GAGGCACGGG AGATTTCTTCATGGCTCGAG GGATTGCCAC GCTTCAGACC GATGATTACG GAGACTTTGT TTATTTCAGGCTCAAGATG

>UdDIR14_contig-7375_CDS

ATGGCAGTGATTAAGAAGAGCTCGGTTTGTCTAATCATAGTCATAATCACAGCTCTCACACTCTTCACGGCAAGCCCATCCTCAGCCCACCCAAACCACAACAAGGGCAATAAGCCACGTCATCGTCGTCCTCCTCAGCCATGCAAAACCCTAGTCCTGTTCTTCCACGACGTGATCTACAACGGTAAGAACGCGAAGAACGCCACGTCGGCGATCGTGGGCGGGCCCGAGGGGTCGAACCGGACGATCCTGGCCGGCCAATTCCACTTCGGCAACGTGGTGGTCTTCGACGACCCGATCACGTTGACCAACGATTTCCACGCTCGTCCCGTGGGCCGAGCCCAAGGGATGTATATATACGACACGAAGAACACGTACACGGCGTGGCTGGGCTTCACGTTCTCGCTCGATAGCCCGGCCCACGGACGAGGGACGATCAGCTTCATCGGAGCCGACCCGCTGATGAACAAGACCAGAGACGTCTCGGTCGTCGGAGGCACCGGAGACTTTCTCATGCACCGCGGAATCGCGACCATCTCGACGGACTCTTTCGAGGGCGAGGTCTATTTCAGGCTCAAGGTTGATATCAAGTTCTACGATTGTTGGACG

>UdPLR1_contig_26577_CDS

ATGGAAATGAGGCAATGTGAAGAGAAAAGCAAAGTGTTGATCGTGGGAGCGACAGGGTATTTGGGGAAGAGGTTAGCGAGGGCGTGTCTGGACGAGGGACACGAGACGTATGTGGTTCATTGGCCCGAGATCGGAGTGGACATCGAGAAAGTTCAGACGCTGTTGGAGTTCAAGGAGAGAGGGGCTCGGCTCGTGACCGCCTCTTTCGAAGACAAAGAGAGCCTCGTGGCCGCCGTGAAGCTCGTCGACGTCGTTATCTGCGCCGTCTCCGGTGTCCATATCCGAACCCATCACATCCTTCTCCAACTCGTTCTCGTCGACGCCATCAAAGAAGCTGGTAACATCAAGAGATTTTTGCCGTCTGAGTTTGGAACTGACCCAGCAAGAATGGGAAATGCAATAGAGCCGGGAAGAGTGACATTTGATGACAAAATGAGAGTGAGAAAAGCAATTGAAGAAGCCAATATTCCTTTCACTTATATCTCTGCCAATTGCTTCGCTGGCTATTTCCTTGGTGGCCTCTGCCAACCTGGTTTCATTCTTCCCTCTACCCAGTCCGTCACTTTGCATGGACATGGCGACGTAAAAGCAATATATGTGGATGAAGATGACATAGCAAGGTACACTATTAAAACCATAGATGACCCTCGAACCCTCAACAAGACAGTGTACATAAGGCCACCCAAAAATATTTTATCACAAAGAGAAGTTGTTCAAGTTTGGGAAAATATCATTGGAAAAGAGTTACACAAGTCTTCAATGTCAAAGGAAGAGTTTTTAGCAACTTTGAAAGAGCAAAATTACGCGGAACAAGTCGGGTTGGGACATTACTACCATGTCTGTTACGAGGGCTGTCTAACCAACTTTGAAATAGGCGAGGAAGGTCTTGAAGCCACCGTGCTTTATCCAGAAGTGAAGTACATT

>UdPLR2_1contig_628_CDS

ATGGGTAAGAGCAAGGTTCTGGTGGTTGGTGGTACAGGGTACATAGGGAAGAGGATAGTTATGGCAAGCCTGGAACAAGGTCACGAAACCTACGTTCTCCGGCGAGCAGAGATCGGTCTCGACATCGACAAGCTTCAGACTCTCTTCTCCTTCAAGAAGCTCGGTGCCCACCTCGTCGAAGGTTCGTTTTCCGACCGCCAAAGCCTCGTCGCCGCCGTGAAACTCGCCGACGTCGTTATCTGTACCATATCCGGCGTTCATTTCCGGAGCCATAATATTCTGCTTCAGCTTGAGCTCGTCGAAGCCATTAAAGAAGCCGGAAATGTCAAGCGCTTTTTGCCGTCGGAGTTTGGTATGGATCCGGCAAAAATGGGACATGCCCTAGAACCAGGAAAAGTGACATTTGACGAGAAAATGACAATTAGGAAAGCGATTGAAGACGCCAATATCCCCTTCACTTATATTTCCGCAAATTGCTTTGCTGGTTATTTCGCCGGAAATCTCGGCCAAATGCAATCTCTTCTTCCTCCCAAGGAAAAAGTTTTTATATATGGAGATGGCAATGCAAAAGTAATTTTGATGGACGAAGATGACGTGGCAACATACACAATCAAAACAATCGACGACCCTCGGACACTGAACAAAACGGTGTACCTTAGACCACAAAGCAACATAATAACGCAGCGACAATTGGTCGAGAAATGGGAAAACATGACCGGAAATAAATTGGAAAAAATTACAATTTCTGACCAAGACTTCCTTGACTCCATTAAAGATTTGGATTATGCACAGCAAGTGGGAGTTGGGCACTTTTATCATGTATTTTACGAAGGTTGTTTGACCAACTTTGAAATTGGAGAAGACGGAGAAGAAGCTTCACAACTCTATCCAGAGGTCAACTACACCACTATGGATCAATACTTGAAAATTTATTTA

>UdPLR3_contig_10583_CDS

ATGGAGGAGCAGAAGCAGAAGAATAGAATTCTGATCGTCGGAGCGACGGGGCGTCTGGGAAGGCATTTGGCCGAGTTCAGCCTCCGATCATCTCATCCCACCTTCGCTCTCGGTAGGTCATCTTCCTTCTCCCTCGCCGCCGATTCACTCCGATCCCTCTCCGCCGCCGGACTTACCATCCTCAAGGGTTCTCTGGAGGACGAGCAGAGTCTGGTCGAGGCCGAGAAGCAAGTGGATGTCGTAATCTCCGCAGTACCGAGCAAGGAAGCTCTCTCTCAGAAGCTTCTGATCAAAGTCATCAACCAATCTGGTTCGATCAAGAGGTTTATTCCGTCTGAATTCGGAGTTGATCCAGATCGGGCTCGAGTTTCCGATATTGATGGCGGTTTCTACTCGAGAAAAGCCAAGATTCGCCGCCTGATCGAATCCCTGGGAATTCCCTACACTTATATATGCTGCAACTACTTCATGAGGATGTTCCTTCCCTCGCTCGTCCAGCCCGGTCTGAGCTCTCCTCCGAGGGACTGTGTCACTATCTTTGGCGACGGAACCGCCAAAGGCGTGTTTGTGAGCGAGAGAGACGTGGCTGCATTCACGATAATGGCGGTGGACGACCCGCGAGCGTTGAACAAGGTGGTGTATTTGAGGCCTCCGGGGAATGTGTATTCGATGAACGAGCTGGTCGGGATTTGGGAGGGCAAGATTGGGAAGAAGCTTGAAAAGGTTTTCGTTTCGGAGCAAGAGCTTCTTCGGAGGATTCACGAGACCCAATATCCTCAGAAAATGGAGATGGTTTTTGTGTACTCGGCTTTCGTGAAAGGAGACCAAACCTACTTTGAAATCGAGGCCTTTGGCGGGGTAGATGGGACCAAGCTGTACCCAGAAGTGAGGTACACAACCATAAGTGAATTTTTAGACACGCTTGTG

>UdDIR1_contig-12966

MIMKTLIIKAITILSLLALASSNDQKYHKSVSPKALGLHKREKLTHIHFYFHDVVAGKNATVVHVAGPPVGLGGPRESFGSVVVMDDPLTVGPDPGSKRIGSAQGIYSSASQTELGFLMVLNYVFTDGKFKGSTLSILGRNAILDEVREMPVVGGTGVFRFATGFAQARTHVFNTTTLDAIVEYNVYVLHY*

>UdDIR2_contig-14063

MVRRTQKTLLSSLITILFLFSISSSHANKYHKTLNPRTLGLRREKLSHLKFYFHDVVSGKSPTVVRVASSPTTNTSPTSFGFVAVMDDPLTLGPEINSTRVGSAQGIYASASQTELGLLMVHTYAFTAGKYNGSSLSILGRNAVFNAVRELPIVGGTGLFRFARGYALAKTHVFDGKSGDAVVKYNVYVLHY*

>UdDIR3_contig-22204

MRTISSNWGFIFLSLLAIIALFYSQPISAANKKRPISPCKRFVVHYHDVMFNGTNSVNATAAVVVNDTALSQTKFGKFVVFDDKMTYDENLSSPEAARAQGFYIYDIKESYGAWFAFSLIFNSTEHKGTLNLLGADLMAEKTRDLSVVGGTGDFFMARGIASVETDSFEGFRYFRLKMDIKLYECY*

>UdDIR4_contig-23037

MVQKISENSRISLCISVALAVLVVVLLAVFTPKLHNSSPSQNAAEWLDLSLYIQEPPRGAHGGDPXAAAAAVAADQADAGALVFHRTLTEGPENTSRIVGKAQ

>UdDIR5_contig-24527

MASNSIISISLLFLVLVLLISPGSNATPNKKSTRHRGRHGINVIKPCKRMVFYFHDIIYNGHNLKNATSSIVGEPAWGNKTILASQNHFGNVVVFDDPITLDNDLHSAPVGRAQGFYLYDKRDVFTSWLGFSFVFDSEEHKGSINFAGADPLMNKTRDISVIGGTGDFFMARGIATLMTDAFEGEVYFRLRVDIKLYECWV*

>UdDIR6_contig-24857

MAKLIISFTSMIMITMMIISSTNAEFARKEEGVTHLEFYFHDTLSGEAPSAVRVADAGEITNKSLTVFGAVLMADDPLTETPDPGSKLVGRAQGLYGSAGQHELALIMAMNFGFIEGEYNGSSISILGKNSAMSPVREMAVVGGTGVFRMARGYAIAHTHWFNLLGDAIVGYNVTIVH*

>UdDIR7_contig-28042

FYFHDVVSGKNPTAVRVAEAPTTNTSATLFGAIVIIDDPLTLGPDPKSPRLGSAQGFYSTVSQSDVVLLMVQNYVFTAGKFNGSTLSVLGRNAPFAAVRELPVVGGTGRFRFARGFALAKTHFYNATTGDAIVQYNVYVNHYY

>UdDIR8_contig-28614

MSSSSIYLIALIVFTANAGFPVLGIGGETVLEMYMHDIVGGNSPTARPVTGLLGNIYSSQVPFAKPIGFTVPDNAVALPNANGALPTVNGVTGLPLGSGLSGTAFAGQVSIQPNSQVSAVQTQLAADGLGLGFGT

>UdDIR9_contig-28699

MAKKLTVTTILSLALLLSVSSVLSSSTTKSKSREKLIMKMGLKKQKLSHFKFFWHDKYSGKNPTAVTIVAPPNDTSFFGSLTMIDDALTERADPNSRLLGRAQGLYGSASRGQVALIMAMNFHFVQGKYNGSGITILGRNPVFDKVREMPVIAGTGLFRFARGFARASTVSFDLATGDAVVQYNVYVLHYG*

>UdDIR10_contig-32790

IYASASQSDLSLLEIFNYVFTDGEFKGSSLSLLGRNAILEEVREMPILGGTGVFRFCRGYALLKTNMFNTTSGDAIVEYDVYVLH

>UdDIR11_contig-34554

MTILFILSLISTSFFISTNGGFLSESKALNKTEKLSHFHFYFHDVLSGKNPSAIKIINPPIPKAGLGLFGETFMIDDALTVGPDPKSKTVGRAQGLYSIATQKSIALLMVMNFAFVDGKYNGSCVSVLGRNSVFDDVRELPIVGGTGSFRFARGYALAHT

>UdDIR12_contig-34733

KPSSSVFLSILLFLVIVLITSESCSASNINNKKPYIKESSPCKRFVLYYHDVLYNGTDLANATSATIANKTALGPYNFGKFVVFNDKLTRDNKLRSPEVARAHGFFFYDMKTTYNAWFAFTLVFNSSDYKGTLNVMGADMMSEKTRDLSVVGGTGDFFMTRGIVTIQTDTYQGDFYFRLKMDIKLYECY*

>UdDIR13_contig-34949

MRTSKPSSSFFFPIILIFVILLITSESCFAEKKPYIKESSPCKRFVLYYHDIHFNGTNAANATSVTASKDNPFGGPYFFGRLVVFDDKMTRDNKPDSPSVARAQGFYFYDMKTKYNAWFAYTLVFDSSEHKGSLNIMGANTISEKTRDLSVVGGTGDFFMARGIATLQTDDYGDFVYFRLKMD

>UdDIR14_contig-7375

MAVIKKSSVCLIIVIITALTLFTASPSSAHPNHNKGNKPRHRRPPQPCKTLVLFFHDVIYNGKNAKNATSAIVGGPEGSNRTILAGQFHFGNVVVFDDPITLTNDFHARPVGRAQGMYIYDTKNTYTAWLGFTFSLDSPAHGRGTISFIGADPLMNKTRDVSVVGGTGDFLMHRGIATISTDSFEGEVYFRLKVDIKFYDCWT*

>UdPLR1_contig_26577

MEMRQCEEKSKVLIVGATGYLGKRLARACLDEGHETYVVHWPEIGVDIEKVQTLLEFKERGARLVTASFEDKESLVAAVKLVDVVICAVSGVHIRTHHILLQLVLVDAIKEAGNIKRFLPSEFGTDPARMGNAIEPGRVTFDDKMRVRKAIEEANIPFTYISANCFAGYFLGGLCQPGFILPSTQSVTLHGHGDVKAIYVDEDDIARYTIKTIDDPRTLNKTVYIRPPKNILSQREVVQVWENIIGKELHKSSMSKEEFLATLKEQNYAEQVGLGHYYHVCYEGCLTNFEIGEEGLEATVLYPEVKYI

>UdPLR2_1contig_628

MGKSKVLVVGGTGYIGKRIVMASLEQGHETYVLRRAEIGLDIDKLQTLFSFKKLGAHLVEGSFSDRQSLVAAVKLADVVICTISGVHFRSHNILLQLELVEAIKEAGNVKRFLPSEFGMDPAKMGHALEPGKVTFDEKMTIRKAIEDANIPFTYISANCFAGYFAGNLGQMQSLLPPKEKVFIYGDGNAKVILMDEDDVATYTIKTIDDPRTLNKTVYLRPQSNIITQRQLVEKWENMTGNKLEKITISDQDFLDSIKDLDYAQQVGVGHFYHVFYEGCLTNFEIGEDGEEASQLYPEVNYTTMDQYLKIYL

>UdPLR3_contig_10583

MEEQKQKNRILIVGATGRLGRHLAEFSLRSSHPTFALGRSSSFSLAADSLRSLSAAGLTILKGSLEDEQSLVEAEKQVDVVISAVPSKEALSQKLLIKVINQSGSIKRFIPSEFGVDPDRARVSDIDGGFYSRKAKIRRLIESLGIPYTYICCNYFMRMFLPSLVQPGLSSPPRDCVTIFGDGTAKGVFVSERDVAAFTIMAVDDPRALNKVVYLRPPGNVYSMNELVGIWEGKIGKKLEKVFVSEQELLRRIHETQYPQKMEMVFVYSAFVKGDQTYFEIEAFGGVDGTKLYPEVRYTTISEFLDTLV
