# Supplementary material for: Insights into Lignan Composition and Biosynthesis in Stinging Nettle (Urtica dioica L.)
Source: Molecules. 2019 Oct 26;24(21):3863. doi: 10.3390/molecules24213863 (PMC6864805; doi:10.3390/molecules24213863)
Supplement: Supplementary file 1 [file molecules-24-03863-s001.zip › Table S3.docx]

**Table S3.** The RPKM value of *UdDIR*s and *UdPLR*s in different tissues obtained from our previous transcriptomic analysis [1].

| Nomenclature | Transcript ID | RPKM | | | |
| --- | --- | --- | --- | --- | --- |
|  |  | **TOP** | **MID** | **BOT-C** | **BOT-F** |
| UdDIR1^*^ | contig_12966 | 1.78 | 2.11 | 1.46 | 10.40 |
| UdDIR2^*^ | contig_14063 | 31.58 | 27.52 | 18.84 | 32.33 |
| UdDIR3 | contig_22204 | 0.00 | 0.00 | 0.09 | 0.66 |
| UdDIR4 | contig_23037 | 1.47 | 0.56 | 1.26 | 0.83 |
| UdDIR5^*^ | contig_24527 | 8.83 | 10.39 | 38.33 | 2.09 |
| UdDIR6 | contig_24857 | 0.00 | 0.00 | 0.06 | 0.05 |
| UdDIR7^*^ | contig_28042 | 0.00 | 0.45 | 15.05 | 0.22 |
| UdDIR8 | contig_28614 | 0.37 | 0.28 | 0.14 | 0.49 |
| UdDIR9^*^ | contig_28699 | 16.80 | 16.51 | 63.63 | 6.42 |
| UdDIR10 | contig_32790 | 0.00 | 0.00 | 0.13 | 0.00 |
| UdDIR11^*^ | contig_34554 | 4.27 | 4.47 | 23.39 | 1.51 |
| UdDIR12^*^ | contig_34733 | 1.03 | 1.32 | 1.95 | 6.90 |
| UdDIR13^*^ | contig_34949 | 11.14 | 10.01 | 0.00 | 0.00 |
| UdDIR14 | contig_7375 | 0.00 | 0.00 | 0.92 | 0.24 |
| UdPLR1^*^ | contig_26577 | 16.57 | 20.63 | 9.16 | 1.17 |
| UdPLR2^*^ | contig_628 | 45.79 | 64.14 | 122.01 | 32.78 |
| UdPLR3^*^ | contig_10583 | 19.62 | 17.15 | 17.49 | 16.38 |

* Differentially expressed genes selected for the RT-qPCR analysis. Differentially expressed genes were determined using *p* < 0.05, minimum value of the means > 1 RPKM and fold change > 4.

**Reference**

1. Xu, X.; Backes, A.; Legay, S.; Berni, R.; Faleri, C.; Gatti, E.; Hausman, J.F.; Cai, G.; Guerriero, G. Cell wall composition and transcriptomics in stem tissues of stinging nettle (Urtica dioica L.): Spotlight on a neglected fibre crop. *Plant Direct* **2019**, *3*, e00151.
