# Supplementary material for: Insights into Lignan Composition and Biosynthesis in Stinging Nettle (Urtica dioica L.)
Source: Molecules. 2019 Oct 26;24(21):3863. doi: 10.3390/molecules24213863 (PMC6864805; doi:10.3390/molecules24213863)
Supplement: Supplementary file 1 [file molecules-24-03863-s001.zip › Table S4.docx]

**Table S4.** Amino acid sequence homology between DIR22 from *Glycine max* (accession number: ADX66343.1) and UdDIRs from subfamily-b/d. The alignment was performed using BLASTP at NCBI.

| Genes | Total score | Query cover (%) | E value | Identity (%) |
| --- | --- | --- | --- | --- |
| UdDIR1 | 156 | 100 | 1E^-52^ | 38.62 |
| UdDIR2 | 160 | 85 | 3E^-54^ | 45.62 |
| UdDIR6 | 112 | 70 | 1E^-35^ | 39.55 |
| UdDIR7 | 124 | 73 | 8E^-41^ | 41.73 |
| UdDIR9 | 145 | 81 | 1E^-48^ | 44.81 |
| UdDIR10 | 81.3 | 44 | 1E^-24^ | 40.96 |
| UdDIR11 | 149 | 76 | 4E^-43^ | 45.99 |
