# Supplementary material for: Insights into Lignan Composition and Biosynthesis in Stinging Nettle (Urtica dioica L.)
Source: Molecules. 2019 Oct 26;24(21):3863. doi: 10.3390/molecules24213863 (PMC6864805; doi:10.3390/molecules24213863)
Supplement: Supplementary file 1 [file molecules-24-03863-s001.zip › Table S2.docx]

**Table S2.** List of the pinoresinol-lariciresinol reductase (PLR) protein accession numbers and amino acid length from different plant species used in this study.

| Species | Nomenclature | Accession number | Length (aa) |
| --- | --- | --- | --- |
| [*Amaranthus hypochondriacus*](https://en.wikipedia.org/wiki/Amaranthus_hypochondriacus) | AmhPLR1 | AHYPO_008142-RA | 313 |
| [*Amborella trichopoda*](https://en.wikipedia.org/wiki/Amborella) | AmtPLR1 | evm_27.model.AmTr_v1.0_scaffold00023.61 | 312 |
|  | AmtPLR2 | evm_27.model.AmTr_v1.0_scaffold00048.121 | 316 |
| *Anacardium occidentale** | AoPLR1 | Anaoc.0014s1052.1 | 314 |
|  | AoPLR2 | Anaoc.0006s0222.1 | 300 |
|  | AoPLR3 | Anaoc.0014s1061.1 | 308 |
|  | AoPLR4 | Anaoc.0014s1063.1 | 291 |
| *Ananas comosus* | AncPLR1 | Aco005975.1 | 312 |
| *Aquilegia coerulea* | AcPLR1 | Aqcoe1G202400.1 | 307 |
|  | AcPLR2 | Aqcoe1G200800.1. | 313 |
|  | AcPLR3 | Aqcoe1G202800.1 | 307 |
| *Arabidopsis halleri* | AhPLR1 | Araha.12896s0005.1 | 317 |
| *Arabidopsis lyrata* | AlPLR1 | AL1G46170.t1 | 317 |
|  | AlPLR2 | AL7G42870.t1 | 317 |
| [*Arabidopsis thaliana*](https://en.wikipedia.org/wiki/Arabidopsis_thaliana) | AtPrR1*^#^* | AT1G32100.1 | 317 |
|  | AtPrR2*^#^* | AT4G13660.1 | 317 |
| *Boechera stricta* | BsPLR1 | Bostr.3359s0148.1 | 318 |
|  | BsPLR2 | Bostr.2021s0127.1 | 346 |
| [*Brachypodium distachyon*](https://en.wikipedia.org/wiki/Brachypodium_distachyon) | BdPLR1 | Bradi4g39260.1 | 307 |
| *Brachypodium stacei* | BrsPLR1 | Brast05G230200.2 | 307 |
|  | BrsPLR2 | Brast05G230300.1 | 307 |
| *Brassica rapa* | BrPLR1 | Brara.E01741.1 | 279 |
|  | BrPLR2 | Brara.I02648.1 | 317 |
| *Capsella grandiflora* | CgPLR1 | Cagra.3359s0007.1 | 459 |
|  | CgPLR2 | Cagra.4326s0015.1 | 322 |
| *Capsella rubella* | CrPLR1 | Carubv10009780m | 318 |
|  | CrPLR2 | Carubv10006801m | 322 |
|  | CrPLR3 | Carubv10009778m | 318 |
| *Carica papaya* | CpPLR1 | evm.model.supercontig_238.6 | 312 |
| *Camellia sinensis* | CasPLR1  CasPLR2 | AYW00065.1  AYW00066.1 | 312  312 |
| *Chenopodium quinoa** | CqPLR1 | AUR62027331-RA | 311 |
|  | CqPLR2 | AUR62007401-RA | 311 |
| *Citrus clementina* | CcPLR1 | Ciclev10001913m | 312 |
|  | CcPLR2 | Ciclev10001909m | 312 |
| *Citrus sinensis* | CisPLR1 | orange1.1g021470m | 312 |
| *Coffea arabica** | CaPLR1 | evm.model.Scaffold_449.254 | 616 |
|  | CaPLR2 | evm.model.Scaffold_449.255 | 312 |
|  | CaPLR3 | evm.model.Scaffold_562.100 | 312 |
|  | CaPLR4 | evm.model.Scaffold_449.258 | 312 |
|  | CaPLR5 | evm.model.Scaffold_562.101 | 312 |
|  | CaPLR6 | evm.model.Scaffold_562.99 | 551 |
| *Cucumis sativus* | CsPLR1 | Cucsa.149830.1 | 309 |
|  | CsPLR2 | Cucsa.149840.1 | 312 |
| [*Daucus carota*](https://en.wikipedia.org/wiki/Daucus_carota) | DcPLR1 | DCAR_008190 | 313 |
|  | DcPLR2 | DCAR_004250 | 312 |
|  | DcPLR3 | DCAR_004251 | 312 |
| [*Eucalyptus grandis*](https://en.wikipedia.org/wiki/Eucalyptus_grandis) | EgPLR1 | Eucgr.E01255.1 | 313 |
|  | EgPLR2 | Eucgr.E01250.1 | 312 |
|  | EgPLR3 | Eucgr.E01253.1 | 312 |
|  | EgPLR4 | Eucgr.E01252.1 | 312 |
| *Eutrema salsugineum* | EsPLR1 | Thhalv10008239m | 317 |
|  | EsPLR2 | Thhalv10009995m | 317 |
|  | EsPLR3 | Thhalv10026944m | 301 |
| *Forsythia intermedia* | FiPLR1*^#^* | AAC49608 | 312 |
| *Fragaria vesca* | FvPLR1 | mrna13540.1-v1.0-hybrid | 806 |
|  | FvPLR2 | mrna13541.1-v1.0-hybrid | 421 |
| *Glycine max* | GmPLR1 | Glyma.09G211500.1 | 318 |
|  | GmPLR2 | Glyma.09G211600.1 | 312 |
|  | GmPLR3 | Glyma.16G103900.1 | 312 |
| *Gossypium hirsutum** | GhPLR1 | Gohir.A08G151300.1 | 313 |
|  | GhPLR2 | Gohir.D08G172700.1 | 312 |
|  | GhPLR3 | Gohir.D08G172500.1 | 313 |
|  | GhPLR4 | Gohir.A08G151600.1 | 312 |
| *Gossypium raimondii* | GrPLR1 | Gorai.004G179900.1 | 312 |
|  | GrPLR2 | Gorai.004G179800.1 | 313 |
| *Isatis indigotica* | IiPLR1 | AEA42007 | 317 |
| *Kalanchoe fedtschenkoi* | KfPLR1 | Kaladp0053s0616.1 | 320 |
|  | KfPLR2 | Kaladp0053s0617.1 | 294 |
|  | KfPLR3 | Kaladp0053s0618.1 | 312 |
| *Kalanchoe laxiflora* | KlPLR1 | Kalax.0008s0005.1 | 312 |
|  | KlPLR2 | Kalax.0008s0006.1 | 318 |
|  | KlPLR3 | Kalax.0008s0007.1 | 320 |
|  | KlPLR4 | Kalax.0025s0136.2 | 320 |
|  | KlPLR5 | Kalax.1260s0001.1 | 284 |
|  | KlPLR6 | Kalax.1260s0002.1 | 312 |
| *Linum album* | LaPLR1*^#^* | CAH60857.1 | 326 |
| *Linum corymbulosum* | LcPLR1*^#^* | ABW86959.1 | 315 |
| Linum perenne | LpPLR1*^#^* | ABM68630.1 | 314 |
| *Linum usitatissimum* | LuPLR1^#^ | Lus10012145 | 312 |
|  | LuPLR2^#^ | Lus10007599 | 658 |
|  | LuPLR3 | Lus10010403 | 314 |
|  | LuPLR4 | Lus10012143 | 314 |
|  | LuPLR5 | Lus10012147 | 328 |
| *Malus domestica* | MdPLR1 | MDP0000144181 | 260 |
|  | MdPLR2 | MDP0000601559 | 352 |
| *Manihot esculenta* | MePLR1 | Manes.15G185300.1 | 312 |
|  | MePLR2 | Manes.17G008900.1 | 212 |
|  | MePLR3 | Manes.17G009000.1 | 312 |
|  | MePLR4 | Manes.17G009100.1 | 312 |
| *Medicago truncatula* | MtPLR1 | Medtr8g071130.1 | 312 |
|  | MtPLR2 | Medtr5g027240.1 | 311 |
|  | MtPLR3 | Medtr5g027290.1 | 311 |
| *Mimulus guttatus* | MgPLR1 | Migut.A00575.1 | 314 |
| *Musa acuminata* | MaPLR1 | GSMUA_Achr4T15440_001 | 309 |
| *Nicotiana tabacum* | NtPLR1 | XP_016486017.1 | 313 |
|  | NtPLR2 | XP_016507984.1 | 320 |
| *Oropetium thomaeum* | OtPLR1 | Oropetium_20150105_10609A | 312 |
|  | OtPLR2 | Oropetium_20150105_10610A | 312 |
| *Oryza sativa* | OsPLR1 | LOC_Os12g16220.2 | 314 |
|  | OsPLR2 | LOC_Os12g16410.1 | 314 |
| *Panicum hallii* | PahPLR1 | Pahal.F02202.1 | 312 |
|  | PahPLR2 | Pahal.F02203.1 | 255 |
|  | PahPLR3 | Pahal.F02205.1 | 255 |
| *Panicum virgatum* | PavPLR1 | Pavir.Ca01941.1 | 307 |
|  | PavPLR2 | Pavir.Cb00180.1 | 312 |
|  | PavPLR3 | Pavir.Cb01083.1 | 283 |
|  | PavPLR4 | Pavir.Cb01084.1 | 307 |
|  | PavPLR5 | Pavir.J10499.1 | 272 |
| *Populus deltoides** | PodPLR1 | Podel.01G143300.1 | 312 |
|  | PodPLR2 | Podel.01G143200.1 | 336 |
|  | PodPLR3 | Podel.03G105700.1 | 309 |
| *Populus trichocarpa* | PopPLR1 | Potri.001G133300.1 | 312 |
|  | PopPLR2 | Potri.001G133200.1 | 313 |
|  | PopPLR3 | Potri.003G100200.1 | 309 |
| *Sinopodophyllum hexandrum^#^* | PhPLR | ACF71492.1 | 311 |
| *Prunus persica* | PpPLR1 | Prupe.5G022800.1 | 312 |
|  | PpPLR2 | Prupe.5G022900.1 | 312 |
|  | PpPLR3 | Prupe.5G023000.1 | 312 |
|  | PpPLR4 | Prupe.5G023200.1 | 312 |
|  | PpPLR5 | Prupe.5G023400.1 | 312 |
|  | PpPLR6 | Prupe.5G023600.1 | 230 |
| *Ricinus communis* | RcPLR1 | 29827.m002565 | 312 |
|  | RcPLR2 | 29827.m002566 | 313 |
| *Salix purpurea* | SpPLR1 | SapurV1A.0051s0460.1 | 312 |
|  | SpPLR2 | SapurV1A.0051s0470.1 | 321 |
|  | SpPLR3 | SapurV1A.0063s0370.1 | 312 |
|  | SpPLR4 | SapurV1A.0063s0380.1 | 312 |
|  | SpPLR5 | SapurV1A.0063s0390.1 | 312 |
| *Selaginella moellendorffii* | SmPLR1 | 438560 | 308 |
|  | SmPLR2 | 230858 | 309 |
| *Sesamum indicum* | SinPLR1 | XP_011092596.1 | 314 |
|  | SinPLR2 | XP_011092597.1 | 312 |
| [*Setaria italica*](https://it.wikipedia.org/wiki/Setaria_italica) | SiPLR1 | Seita.3G297700.3 | 255 |
|  | SiPLR2 | Seita.3G394400.1 | 312 |
| *Setaria viridis* | SvPLR1 | Sevir.3G329400.1 | 304 |
|  | SvPLR2 | Sevir.3G412600.1 | 312 |
| *Solanum lycopersicum* | SlPLR1 | Solyc06g066170.2.1 | 313 |
|  | SlPLR2 | Solyc06g066160.2.1 | 311 |
| *Solanum tuberosum* | StPLR1 | PGSC0003DMT400052746 | 313 |
|  | StPLR2 | PGSC0003DMT400052699 | 293 |
| [*Sorghum bicolor*](https://en.wikipedia.org/wiki/Sorghum_bicolor) | SbPLR1 | Sobic.008G106700.1 | 314 |
|  | SbPLR2 | Sobic.008G106800.2 | 312 |
| *Spirodela polyrhiza* | SpipPLR1 | Spipo30G0007500 | 309 |
|  | SpipPLR2 | Spipo30G0007800 | 309 |
|  | SpipPLR3 | Spipo30G0007900 | 321 |
| *Theobroma cacao* | TcPLR1 | Thecc1EG016648t1 | 312 |
|  | TcPLR2 | Thecc1EG016649t1 | 313 |
| *Thuja plicata* | TpPLR1^#^  TpPLR2^#^  TpPLR3  TpPLR4 | AAF63507.1  AAF63508.1  AAF63509.1  AAF63510.1 | 313  312  314  312 |
| *Trifolium pratense* | TrpPLR1 | Tp57577_TGAC_v2_mRNA30123 | 314 |
|  | TrpPLR2 | Tp57577_TGAC_v2_mRNA32373 | 312 |
| *Triticum aestivum** | TaPLR1 | Traes_2DS_3663CF5CF.1 | 313 |
|  | TaPLR2 | Traes_2DS_BCA5E00B5.1 | 313 |
|  | TaPLR3 | Traes_2AS_621B8FE92.1 | 313 |
|  | TaPLR4 | Traes_2BS_23E0AC565.2 | 311 |
|  | TaPLR5 | Traes_7AS_94BEBA950.1 | 307 |
|  | TaPLR6 | Traes_2AS_8BC8B373D.1 | 240 |
| *Vitis vinifera* | VvPLR1 | GSVIVT01029357001 | 312 |
|  | VvPLR2 | GSVIVT01025822001 | 266 |
|  | VvPLR3 | GSVIVT01022640001 | 218 |
|  | VvPLR4 | GSVIVT01022642001 | 265 |
|  | VvPLR5 | GSVIVT01022644001 | 312 |
|  | VvPLR6 | GSVIVT01025819001 | 331 |
| *Zea mays* | ZmPLR1 | GRMZM2G326116_T01 | 312 |

**Early release genomes; ^#^ Enantiospecificity characterized
