# Supplementary material for: Insights into Lignan Composition and Biosynthesis in Stinging Nettle (Urtica dioica L.)
Source: Molecules. 2019 Oct 26;24(21):3863. doi: 10.3390/molecules24213863 (PMC6864805; doi:10.3390/molecules24213863)
Supplement: Supplementary file 1 [file molecules-24-03863-s001.zip › Table S1.docx]

**Table S1.** List of the dirigent protein (DIR) accession numbers and amino acid length from different plant species used in this study.

| Species | Nomenclature | Accession number | Length (aa) |
| --- | --- | --- | --- |
| *Medicago truncatula* | MtDIR1 | Medtr1g046490 | 236 |
|  | MtDIR2 | Medtr1g046500 | 250 |
|  | MtDIR3 | Medtr1g046800 | 250 |
|  | MtDIR4 | Medtr1g054525 | 192 |
|  | MtDIR5 | Medtr1g056370 | 186 |
|  | MtDIR6 | Medtr1g115510 | 353 |
|  | MtDIR7 | Medtr1g115515 | 236 |
|  | MtDIR8 | Medtr3g034030 | 186 |
|  | MtDIR9 | Medtr3g105630 | 189 |
|  | MtDIR10 | Medtr3g105640 | 188 |
|  | MtDIR11 | Medtr4g013310 | 197 |
|  | MtDIR12 | Medtr4g013320 | 199 |
|  | MtDIR13 | Medtr4g013325 | 199 |
|  | MtDIR14 | Medtr4g013330 | 199 |
|  | MtDIR15 | Medtr4g013335 | 196 |
|  | MtDIR16 | Medtr4g013345 | 196 |
|  | MtDIR17 | Medtr4g013350 | 197 |
|  | MtDIR18 | Medtr4g013355 | 180 |
|  | MtDIR19 | Medtr4g013385 | 191 |
|  | MtDIR20 | Medtr4g013770 | 192 |
|  | MtDIR21 | Medtr4g049550 | 179 |
|  | MtDIR22 | Medtr4g049570 | 178 |
|  | MtDIR23 | Medtr4g062460 | 257 |
|  | MtDIR24 | Medtr4g062470 | 304 |
|  | MtDIR25 | Medtr4g062520 | 303 |
|  | MtDIR26 | Medtr4g073950 | 192 |
|  | MtDIR27 | Medtr4g074020 | 99 |
|  | MtDIR28 | Medtr4g078885 | 186 |
|  | MtDIR29 | Medtr4g122110 | 180 |
|  | MtDIR30 | Medtr4g122130 | 180 |
|  | MtDIR31 | Medtr5g096120 | 189 |
|  | MtDIR32 | Medtr7g021300 | 389 |
|  | MtDIR33 | Medtr7g070390 | 192 |
|  | MtDIR34 | Medtr7g093790 | 191 |
|  | MtDIR35 | Medtr7g093820 | 194 |
|  | MtDIR36 | Medtr7g093830 | 193 |
|  | MtDIR37 | Medtr7g093850 | 194 |
|  | MtDIR38 | Medtr7g093870 | 218 |
|  | MtDIR39 | Medtr8g073770 | 190 |
|  | MtDIR40 | Medtr8g073850 | 190 |
|  | MtDIR41 | Medtr8g099115 | 189 |
|  | MtDIR42 | Medtr8g099135 | 185 |
| *Oryza sativa* | OsDIR1 | Os07g0107300 | 229 |
|  | OsDIR2 | Os07g0107500 | 220 |
|  | OsDIR3 | Os07g0107100 | 209 |
|  | OsDIR4 | Os08g0375400 | 207 |
|  | OsDIR5 | Os10g0398100 | 188 |
|  | OsDIR6 | Os07g0617500 | 189 |
|  | OsDIR7 | Os07g0617100 | 175 |
|  | OsDIR8 | BAB89617 | 150 |
|  | OsDIR9 | Os07g0106900 | 204 |
|  | OsDIR10 | Os03g0400200 | 177 |
|  | OsDIR11 | Os07g0636600 | 204 |
|  | OsDIR12 | Os07g0636800 | 202 |
|  | OsDIR13 | Os07g0637700 | 229 |
|  | OsDIR14 | BAC19943 | 207 |
|  | OsDIR15 | Os07g0638500 | 205 |
|  | OsDIR16 | BAC16397 | 154 |
|  | OsDIR17 | AAM74352 | 204 |
|  | OsDIR18 | BAD25846 | 149 |
|  | OsDIR19 | Os03g0809000 | 186 |
|  | OsDIR20 | AAO17346 | 275 |
|  | OsDIR21 | BAB64642 | 340 |
|  | OsDIR22 | BAD52647 | 376 |
|  | OsDIR23 | Os10g0398600 | 38 |
|  | OsDIR24 | BAD53304 | 169 |
|  | OsDIR25 | AAM74358 | 175 |
|  | OsDIR26 | AAM74346 | 191 |
|  | OsDIR27 | BAD03849 | 188 |
|  | OsDIR28 | BAD03720 | 188 |
|  | OsDIR29 | BAD03711 | 188 |
|  | OsDIR30 | BAD03854 | 144 |
|  | OsDIR31 | Os12g0174700 | 184 |
|  | OsDIR32 | Os11g0180000 | 185 |
|  | OsDIR33 | Os11g0178800 | 176 |
|  | OsDIR34 | AAX96293 | 184 |
|  | OsDIR35 | Os11g0179100 | 177 |
|  | OsDIR36 | Os11g0179000 | 178 |
|  | OsDIR37 | Os12g0198700 | 307 |
|  | OsDIR38 | Os12g0247700 | 306 |
|  | OsDIR39 | Os12g0228700 | 247 |
|  | OsDIR40 | Os12g0199000 | 258 |
|  | OsDIR41 | Os12g0227500 | 154 |
|  | OsDIR42 | ABA94701 | 296 |
|  | OsDIR43 | Os11g0644700 | 112 |
|  | OsDIR44 | BAB89759 | 249 |
|  | OsDIR45 | Os11g0645400 | 170 |
|  | OsDIR46 | Os04g0666800 | 179 |
|  | OsDIR47 | Os11g0214400 | 192 |
|  | OsDIR48 | Os11g0214900 | 189 |
|  | OsDIR49 | Os11g0215100 | 181 |
|  | OsDIR50 | Os08g0349100 | 188 |
|  | OsDIR51 | BAD89460 | 176 |
|  | OsDIR52 | AAX96290 | 182 |
|  | OsDIR53 | ABA93522 | 160 |
|  | OsDIR54 | AAX96314 | 178 |
| *Arabidopsis thaliana* | AtDIR1 | At5g42510 | 182 |
|  | AtDIR2 | At5g42500 | 185 |
|  | AtDIR3 | At5g49040 | 191 |
|  | AtDIR4 | At2g21110 | 186 |
|  | AtDIR5 | At1g64160 | 182 |
|  | AtDIR6 | At4g23690 | 187 |
|  | AtDIR7 | At3g13650 | 186 |
|  | AtDIR8 | At3g13662 | 173 |
|  | AtDIR9 | At2g39430 | 322 |
|  | AtDIR10 | At2g28670 | 447 |
|  | AtDIR11 | At1g22900 | 187 |
|  | AtDIR12 | At4g11180 | 185 |
|  | AtDIR13 | At4g11190 | 184 |
|  | AtDIR14 | At4g11210 | 184 |
|  | AtDIR15 | At4g38700 | 190 |
|  | AtDIR16 | At3g24020 | 243 |
|  | AtDIR17 | AB67637 | 271 |
|  | AtDIR18 | At4g13580 | 244 |
|  | AtDIR19 | At1g58170 | 185 |
|  | AtDIR20 | At1g55210 | 187 |
|  | AtDIR21 | At1g65870 | 189 |
|  | AtDIR22 | At3g13660 | 125 |
|  | AtDIR23 | At2g21100 | 187 |
|  | AtDIR24 | At3g55230 | 306 |
|  | AtDIR25 | At1g07730 | 459 |
| *Cannabis sativa* | CsaDIR6A | csa_locus_13101_iso_4_len_657_ver_2 | 189 |
|  | CsaDIR6B | csa_locus_18252_iso_1_len_450_ver_2 | 197 |
|  | CsaDLP1c | sa_locus_63472_iso_1_len_673_ver_2 | 137 |
|  | CsaDLP2 | csa_locus_13020_iso_3_len_979_ver_2 | 194 |
|  | CsaDLP3 | EST01417 | 120 |
|  | Csa-DLP4 | csa_locus_19404_iso_1_len_1338_ver_2 | 251 |
|  | CsaDLP5 | csa_locus_61511_iso_1_len_851_ver_2 | 238 |
|  | CsaDLP20A | csa_locus_62520_iso_1_len_631_ver_2 | 126 |
|  | CsaDLP20B | csa_locus_33311_iso_1_len_752_ver_2 | 206 |
| *Linum usitatissimum* | LuDIR1 | Lus10032331 | 163 |
|  | LuDIR2 | Lus10024715 | 161 |
|  | LuDIR3 | Lus10024714 | 161 |
|  | LuDIR5 | Lus10028749 | 168 |
|  | LuDIR6 | Lus10017539 | 166 |
| *Picea glauca** | PDIR1 | ABD52112 | 186 |
|  | PDIR7 | ABD52118 | 188 |
|  | PDIR10 | ABD52121 | 184 |
|  | PDIR12 | ABD52123 | 184 |
|  | PDIR18 | ABD52129 | 224 |
| *Picea sitchensis** | PDIR11 | ABD52122 | 186 |
|  | PDIR15 | ABD52126 | 196 |
|  | PDIR16 | ABD52127 | 196 |
|  | PDIR17 | ABD52128 | 178 |
|  | PDIR19 | ABD52130 | 159 |
|  | PDIR20 | EF643700 | 186 |
|  | PDIR21 | EF643701 | 184 |
|  | PDIR22 | EF643702 | 174 |
|  | PDIR23 | EF643703 | 168 |
|  | PDIR24 | EF643704 | 168 |
|  | PDIR25 | EF643705 | 175 |
|  | PDIR26 | EF643706 | 177 |
|  | PDIR27 | EF643707 | 176 |
|  | PDIR28 | EF643708 | 172 |
|  | PDIR29 | EF643709 | 172 |
|  | PDIR30 | EF643710 | 164 |
|  | PDIR31 | EF643711 | 164 |
|  | PDIR32 | EF643712 | 195 |
|  | PDIR33 | EF643713 | 196 |
|  | PDIR34 | EF643714 | 194 |
|  | PDIR35 | EF643715 | 148 |
| *Picea glauca x engelmannii** | PDIR2 | ABD52113 | 195 |
|  | PDIR3 | ABD52114 | 188 |
|  | PDIR4 | ABD52115 | 184 |
|  | PDIR5 | ABD52116 | 196 |
|  | PDIR6 | ABD52117 | 195 |
|  | PDIR8 | ABD52119 | 196 |
|  | PDIR9 | ABD52120 | 192 |
|  | PDIR13 | ABD52124 | 195 |
|  | PDIR14 | ABD52125 | 184 |
| *Triticum aestivum** | TaDIR1 | AAC49284 | 343 |
|  | TaDIR2 | AAM46813 | 245 |
|  | TaDIR3 | BAA32786 | 300 |
|  | TaDIR4 | AAR20919 | 304 |
|  | HvDIR1 | AAA87042 | 304 |
|  | HvDIR2 | AAA87041 | 304 |
|  | HvDIR3 | AAB72098 | 306 |
| *Isatis indigotica*** | IiDIR1 | comp13356_c0_seq1 | 182 |
|  | IiDIR2 | comp33656_c0_seq1 | 187 |
|  | IiDIR3 | comp22738_c0_seq1 | 187 |
|  | IiDIR4 | comp22738_c0_seq2 | 187 |
|  | IiDIR5 | comp20562_c1_seq1 | 185 |
|  | IiDIR6 | comp20562_c1_seq2 | 185 |
|  | IiDIR7 | comp28741_c0_seq1 | 186 |
|  | IiDIR8 | comp28741_c0_seq2 | 186 |
|  | IiDIR9 | comp29140_c0_seq1 | 186 |
|  | IiDIR10 | comp33689_c0_seq1 | 188 |
|  | IiDIR11 | comp29669_c0_seq1 | 189 |
|  | IiDIR12 | comp32977_c0_seq1 | 190 |
|  | IiDIR13 | comp26249_c0_seq1 | 267 |
|  | IiDIR14 | comp26249_c0_seq2 | 243 |
|  | IiDIR15 | comp26249_c0_seq3 | 267 |
|  | IiDIR16 | comp14231_c0_seq1 | 314 |
|  | IiDIR17 | comp32662_c0_seq1 | 316 |
|  | IiDIR18 | comp18321_c3_seq1 | 223 |
|  | IiDIR19 | comp30687_c1_seq1 | 415 |
| *Gossypium barbadense* | GbDIR1 | AAS73001.2 | 176 |
|  | GbDIR2 | AAY44415.1 | 174 |
| *Nicotiana benthamiana* | NbDIR1 | BAF02555.1 | 194 |
| *Arachis hypogaea* | AhDIR1 | AAZ20288.1 | 203 |
| *Agrostis stolonifera* | AsDIR1 | AAY41607.1 | 319 |
| *Forsythia x intermedia* | FiDIR1 | AAF25357.1 | 186 |
|  | FiDIR2 | AAF25358.1 | 185 |

* Full length cDNA Accession No.; ** Transcription profiling ID
