# Supplementary material for: Insights into Lignan Composition and Biosynthesis in Stinging Nettle (Urtica dioica L.)
Source: Molecules. 2019 Oct 26;24(21):3863. doi: 10.3390/molecules24213863 (PMC6864805; doi:10.3390/molecules24213863)
Supplement: Supplementary file 1 [file molecules-24-03863-s001.zip › Figure S1.docx]

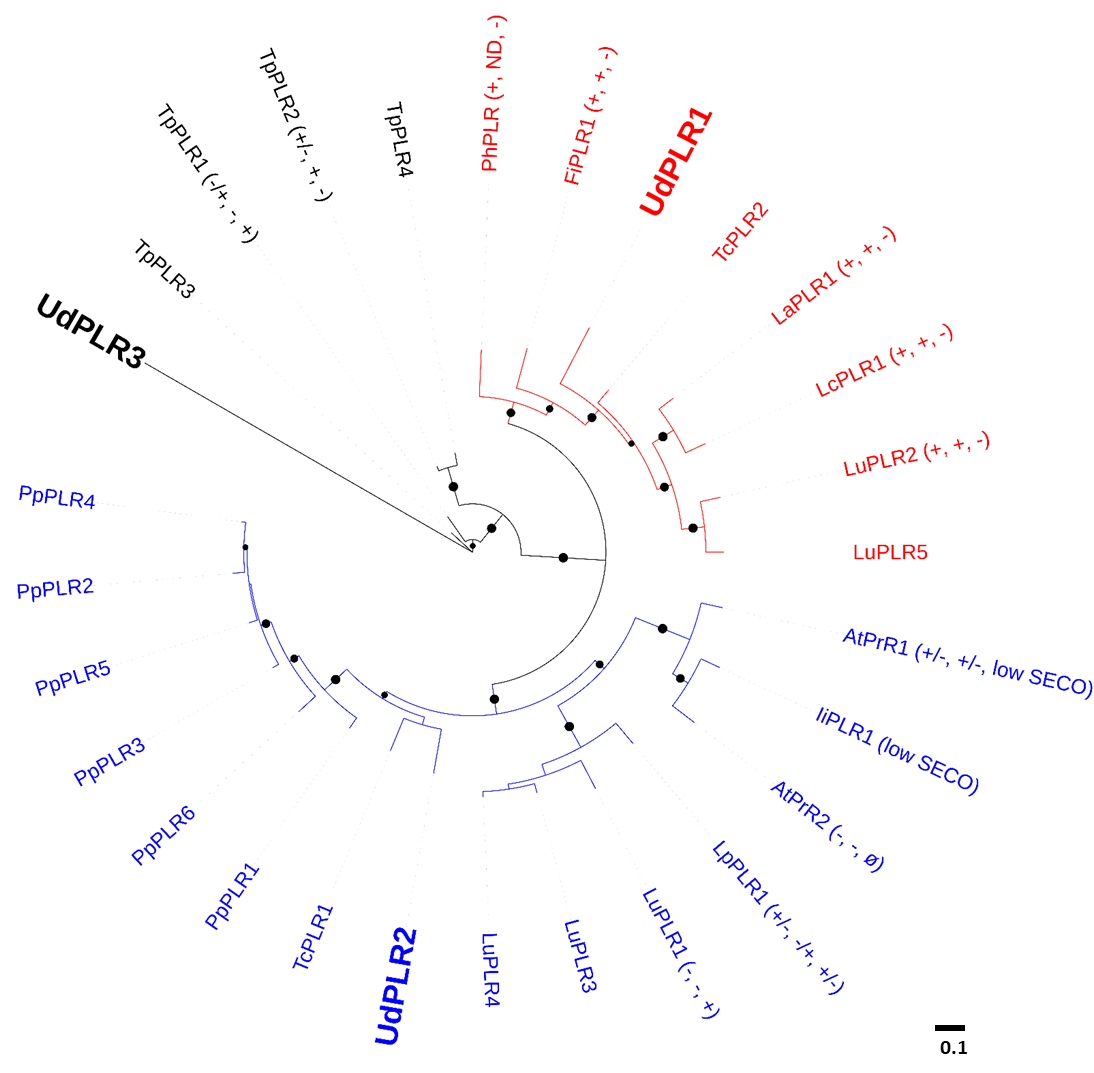


**Figure S1.** Phylogenetic analysis of UdPLRs and PLRs that were characterized for the enantiospecificity. The tree was built by the maximum likelihood method with 1000 bootstraps. Ud, *Urtica dioica*; At, *Arabidopsis thaliana*; Fi, *Forsythia x intermedia*; Lu, *Linum usitatissimum*; La, *Linum album*; Lc, *Linum corymbulosum*; Tp, *Thuja plicata*; Pp, *Prunus persica*; Tc, *Theobroma cacao*. Bootstrap values >80% are indicated with black circles. Enantiospecificity of characterized PLRs are shown in bracket in the order of (PINO, LARI, SECO), + and - represent two different enantiomeric configuration. Ø refers to “not detected”. ND refers to “not determined”. Detailed information of each protein is shown in Table S2. The scale bar indicates 0.1 amino acid substitutions per site.
